# Biochemical and Genetic Approaches Improving Nitrogen Use Efficiency in Cereal Crops: A Review

**DOI:** 10.3389/fpls.2021.657629

**Published:** 2021-06-04

**Authors:** Nitika Sandhu, Mehak Sethi, Aman Kumar, Devpriya Dang, Jasneet Singh, Parveen Chhuneja

**Affiliations:** School of Agricultural Biotechnology, Punjab Agricultural University, Ludhiana, India

**Keywords:** biochemical, cereal, genetic, genes, nitrogen use efficiency, N fertilizer, QTL

## Abstract

Nitrogen is an essential nutrient required in large quantities for the proper growth and development of plants. Nitrogen is the most limiting macronutrient for crop production in most of the world’s agricultural areas. The dynamic nature of nitrogen and its tendency to lose soil and environment systems create a unique and challenging environment for its proper management. Exploiting genetic diversity, developing nutrient efficient novel varieties with better agronomy and crop management practices combined with improved crop genetics have been significant factors behind increased crop production. In this review, we highlight the various biochemical, genetic factors and the regulatory mechanisms controlling the plant nitrogen economy necessary for reducing fertilizer cost and improving nitrogen use efficiency while maintaining an acceptable grain yield.

## Introduction

**FIGURE 1:**
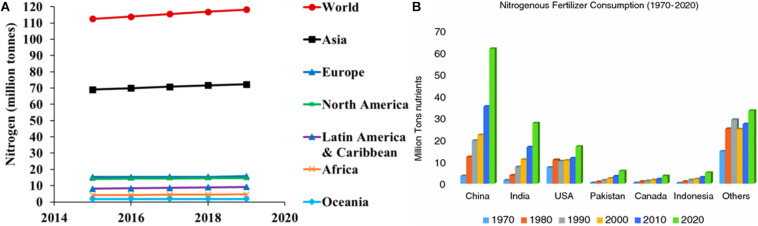
**(A)** The world and regional demand for nitrogen fertilizer forecasts, 2015–2019. Adapted from FAO (2016). **(B)** The consumption of nitrogenous fertilizer from 1970 to 2020 across different countries of the world.

Cereal crops are highly cultivated in comparison to other crops worldwide. Among cereals, rice (*Oryza sativa* L.), wheat (*Triticum aestivum* L.), and maize (*Zea mays* L.) are most important in terms of human nutrition and represent 90% of cereal production worldwide. The value of cereal crops in world agriculture has significantly increased since the Green Revolution. The three major cereal crops are known to address the world protein and calorie demand either directly by human consumption or indirectly through livestock (Ladha et al., 2016; Guerrieri and Cavaletto, 2018). Many factors are known to influence the quality and quantity of cereal crops produced worldwide, and the most important among them is nitrogen availability. All plants depend on the external source of inorganic nitrogen (N), as it is the essential component of biomolecules, including proteins, nucleic acids, chlorophyll, and several secondary metabolites. In agricultural practices, nitrogen availability is a limiting factor to enhance the yield, and worldwide approximately 100 TgNyr^–1^ of reactive nitrogen is applied in the form of fertilizers to crop fields (Ladha et al., 2016). Globally, the total N fertilizer consumption has grown from 112.5 million tons in 2015 to 118.2 million tons in 2019 (see Figure 1A). Between 1970 and 2020, nitrogenous fertilizer consumption has increased at a higher rate across different countries (Figure 1B). It is observed that in cereals, yield can be directly correlated to nitrogen application (Ladha et al., 2016). Approximately 94 million tons of N fertilizer is applied to cereal crops every year, but less than 40% is utilized by the crops, while the remaining part dissipates in the environment, raising severe environmental issues such as water pollution and greenhouse gas emission (Plett et al., 2018). A total of 44 million tons of nitrogen accounts for biological fixation by the legumes and other plants, where 99 million tons accounts for other anthropogenic sources such as habitat destruction and fossil fuels (Matson et al., 2002). Natural sources such as soil bacteria, algae, and lightning account for 154 million tons. Among the cereal crops, barley has maximum nitrogen recovery (63%) followed by maize (37%), wheat (35–45%), and rice (30–50%). Nitrogen recovery not only changes with crop type it also depends on the environmental condition, type of fertilizers used, management strategy, and genotype to environment interactions.

**FIGURE 2:**
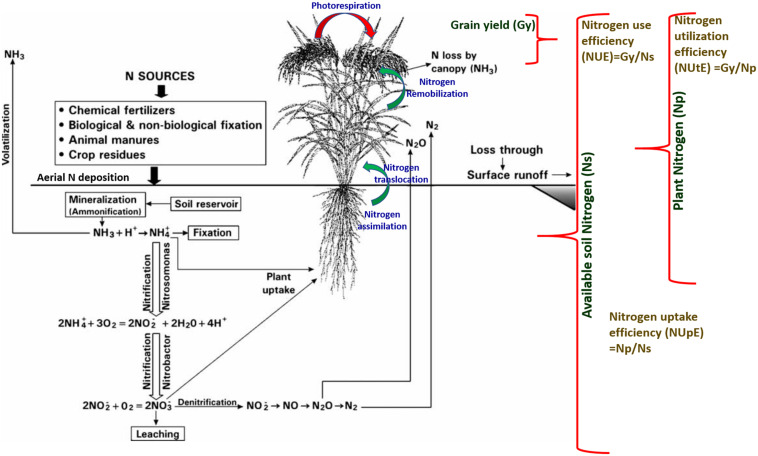
Schematic representation of the relationship between the nitrogen sources, key physiological processes for the nitrogen assimilation, translocation, remobilization, uptake, utilization, and conversion to the grain yield.

In the post-Green Revolution era, traditional varieties were replaced by a few selected and widely adapted semi-dwarf, early-maturing, high-yielding, disease-resistant varieties that require high input conditions. The consumption of fertilizers is expected to double by 2050, i.e., from 112 Mt in 2015 to 236 Mt in 2050 (Tilman et al., 2011). Nevertheless, nitrogen fertilizer utilization is relatively inefficient. Around 50–70% of applied nitrogen always vanishes from the plant-soil system. The high input of commercially available fertilizers has led to the degradation of air, soil, and water quality with the exhaustion of natural resources such as nutrients and water. When the nitrogen supply is more than crop nitrogen demand, it leads to the accumulation of nitrogen in the soil, and the plants are susceptible to various loss pathways. Therefore, it is necessary to improve the resource use efficiency of cereal crops to minimize the negative impact of increasing yield on environments and natural resources. To reduce the effect of the increasing use of fertilizers on climate change and manage sustainable feeding to the growing world population, enhancing nitrogen use efficiency (NUE) in cereals must be a priority in breeding programs. It is essential to understand the underlying mechanism of nitrogen use efficiency to encounter the issues related to nitrogen application in fields. The use of N (nitrogen) in plants involves several stages, which can be divided into the primary N uptake phase, followed by reduction of nitrogen to useable forms, its assimilation into amino acids, translocation, and the last stage is remobilization of nitrogen to reproductive tissues (Figure 2; Masclaux-Daubresse et al., 2010). NUE in cereal crops is defined as the grain yield per unit of nitrogen available in the soil (Moll et al., 1982; Figure 2). The analysis of NUE gives details about plant response to different nitrogen availability conditions. Nitrogen use efficiency can be described by various formulas and definitions. Cereal NUE resulted from the combination between how effectively plants capture the nitrogen (uptake efficiency, NUpE) and how the plants use the taken-up nitrogen (utilization efficiency, NUtE) (Figure 2; Hansen et al., 2018). NUpE is calculated as the total amount of above-ground nitrogen content during harvest by available N in the soil, and NUtE is calculated as the nitrogen in grain tissues divided by N in above-ground plant biomass at harvest (Figure 2). So NUE is calculated at the time of harvest, i.e., end of the crop cycle. The usage index (UI) takes into account the absolute increase in the biomass and can be calculated as UI: shoot weight ^∗^(shoot weight/nitrogen content of the shoot) (Siddiqi and Glass, 1981). Craswell and Godwin (1984) described agronomic efficiency as differences between the grain weight with and without fertilizer divided by the total fertilizer applied; apparent nitrogen recovery as differences between the plant nitrogen uptake with and without fertilizer divided by the total fertilizer applied and multiplied by the factor 100; and the physiological efficiency as the differences between the grain weight with and without fertilizer divided by plant nitrogen uptake with and without fertilizer. The agronomic efficiency measures the efficiency of plants converting the applied nitrogen to the grain yield whereas the apparent nitrogen efficiency of plants captures the nitrogen from the soil (Craswell and Godwin, 1984). The physiological efficiency measures the efficiency of plants in terms of converting the capturing nitrogen to the grain yield.

Improving resource use efficiency to minimize the negative impact of increasing yield with increasing input use on environments/natural resources is an urgent need for major cereal crops. The challenge here is to identify the specific and most responsive stage to the fertilizer application, having a plant that maximizes its early nitrogen uptake, and having traits such as early vegetative vigor and an extensive root system for efficient nutrient uptake considering above and below ground level factors. Later in growth development, a plant with the ability to assimilate and remobilize the available nitrogen and associated carbon to the grain is crucial. Another key challenge here includes appropriate root phenotyping, genotype x environmental interactions, soil characteristics, water-nutrient management, and nutrient dynamics balance. The key question, whether the improvement of nutrient uptake with reducing excessive input of fertilizers and safeguarding soil-health while maintaining the desired yield and grain protein content is feasible. Nanotechnology, including the use of nano fertilizers (1–100 nm in size) is beneficial and reported to have positive results, but still there is a need to specify the effect of nano fertilizers on specific crops (Cowling and Field, 2003).

Before understanding the biochemistry and genetics behind the improvement of nitrogen use efficiency in cereal crops, there is a need to understand the new potential source of nitrogen fertilizers, effect of nitrogen at different stages of growth, nitrogen status of the crop, and development and NUE in the effect of fertilizers (Cameron et al., 2013). The multiple fertilizer sources include anhydrous ammonia (82%N), urea (46%N), ammonium nitrate (34%), ammonium nitrate sulfate (26%), and aqua ammonia (25%N). Nitrogen fertilizers can be broadly classified into organic and inorganic fertilizers. Firstly, looking in terms of inorganic fertilizers, maximum nitrogen, i.e., more than 80%, is contributed by anhydrous ammonia application. Aqua ammonia or ammonium hydroxide is the second most important source of inorganic nitrogen fertilizers and it contains 25 to 29% ammonia by weight. Another form of nitrogen fertilizer is ammonium nitrate and its relevance from the agronomic aspect is a combination of two different forms of nitrogen (NH_4_NO_3_). This form of fertilizer is reported to enhance the baking quality of wheat (Dobermann and Cassman, 2002). Ammonium sulfate ((NH_4_)_2_SO_4_) is an important source of both nitrogen and sulfur that can be advantageous for crops that require acid such as rice and in high-pH soils. Another form of fertilizer that comes with a dual nutrient composition which acts as the source of nitrogen, phosphorous, and chloride include monoammonium (NH_4_H_2_PO_4_), diammonium (NH_4_)_2_HPO_4_) phosphates, ammonium chloride (NH_4_Cl), and ammonium sulfate (Inman et al., 2005). The organic form of fertilizer is urea [CO(NH_2_)_2_] (Franzen et al., 2002).

**FIGURE 3:**
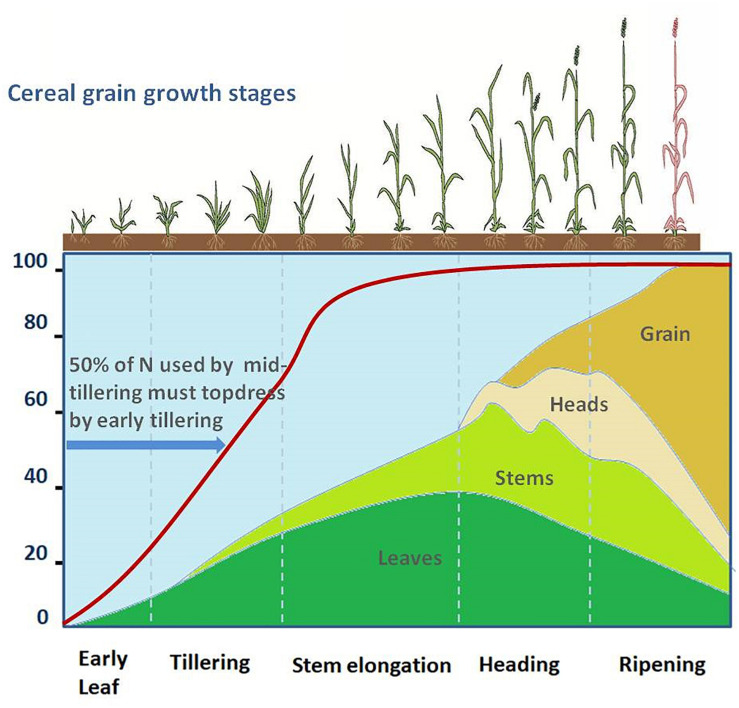
The different stages of growth and development in cereal crops. The red line indicates the requirement of nitrogen at different stages of growth and development, the colored areas under different curves show the accumulation of nitrogen in leaves, stem, head, and grain of spring wheat as a percent of maximum nitrogen. Source: Adapted and modified from Miller et al. (1993).

The cereal crop undergoes different stages of growth and development (Figure 3). The rate of nutrient uptake varies with the crop, crop growth stage, variety, and with growing conditions and environment. Proper understanding of the nutrient uptake patterns of cereal crops is required to determine the optimal timing and specific stage of fertilizer applications. Small amounts of nitrogen are important at an early stage for seedling vigor. About 50% of the required nitrogen is used up by the mid-tillering stage (Miller et al., 1993; Figure 3). However, a high dose of nitrogen may damage the seedlings and over-stimulate the vegetative growth early in the season and thus decrease the yields. Excess nitrogen may delay crop maturity. In the Montana study, more than 70% of the total above-ground N had been accumulated by the beginning of the grain filling stage (Figure 3).

Several factors are reported to influence the requirement of nitrogen and it is observed that NUE decreases when nitrogen application exceeds the potential demand (Mullen et al., 2003). The most active area concerned with the increase in NUE in the crop system is the identification of the nitrogen status of the crop. It is described that there is no linear relation between nitrogen applied and crop yield, as NUE is regulated by several factors (Inman et al., 2007), such as soil type, availability of other macro and micronutrients (phosphorous, potassium, etc.) in soil, and crop rotation reported to regulate the nitrogen uptake and utilization (Hatfield et al., 2008). Nitrogen fertilization also depends on intensity, timing, and depth of tillage (Cassman et al., 2002; Osborne et al., 2002). There is a need to improve strategies to diagnose nitrogen status and this is the most active area of research to enhance the output of N fertilization. Among several N estimation approaches estimation of nitrate and ammonium forms in the soil, satellite imaging (Sharma et al., 2016), portable hyperspectral sensors (Shaver et al., 2014), drones, chlorophyll meters (SPAD), red edge optical reflectance (R750/R710) (Sharma and Franzen, 2013; Sharma et al., 2015), NDVI (normalized vegetation index), and RVI (ration vegetation index) (Sharma et al., 2016) offer the possibility of N estimation in less time.

The wild and primitive species of cereal crops are not fully recognized yet as an important source of novel variations for nutrient utilization efficiency. Association studies exploiting the best alleles to be assembled in superior varieties, identification and characterization of candidate genes with non-synonymous and regulatory SNPs will help breeders to choose specific donors to develop resource-efficient high yielding wheat varieties. Further, yield and grain protein content which represent nitrogen use efficiency are inversely related, so it very important for breeders to design cultivation programs to achieve comparatively successful NUE without compromising grain yield (Oury and Godin, 2007) and to develop such cultivars, it is very important to understand the details of various genetic, physiological, and biochemical factors affecting NUpE and NUtE.

Along-with-it agronomic practices and field management played a major role in combating loss of applied nitrogen to the environment (Karamanos et al., 2014). The present review focuses on the multiple biochemical and genetic factors affecting NUE directly and indirectly. The review gives a descriptive outline about the biochemistry involved in nitrogen uptake and utilization; genetic system influencing NUE among cereals and their phenotype outcomes positively affecting NUE. The related study among cereals is beneficial to design strategies for the combined increase in NUE without affecting other beneficial traits.

## Biochemical and Biological Processes

Several metabolic processes in coordination influence the nitrogen use efficiency of higher plants. It is very important to understand the nitrogen use efficiency and its component before getting into the details of mechanisms affecting efficient NUE. The analysis of NUE gives details about plant response to different nitrogen availability conditions. Nitrogen use efficiency can be bifurcated into two components, i.e., nitrogen uptake efficiency (NUpE) and nitrogen utilization efficiency (NUtE) so to estimate NUE both of its components have to be calculated. NUpE is calculated as the total amount of above-ground nitrogen content during harvest by available N in the soil, and NUtE is calculated as the nitrogen in grain tissues divided by N in above-ground plant biomass at harvest. So NUE is calculated at the time of harvest, i.e., end of the crop cycle. However, yield and grain protein content which represent the nitrogen use efficiency are inversely related, so it very important for breeders to design cultivation programs to achieve comparatively successful NUE without compromising grain yield (Oury and Godin, 2007) and to develop such cultivars, it is very important to understand the details of various traits which affect NUpE and NUtE and keeping this in consideration, processes and traits related to NUE are discussed in detail.

### Traits Affecting Nitrogen Uptake Efficiency

#### Root Architecture

Root development and root system architecture are highly responsive to nutrient availability. To date, the root architectural plasticity traits, genetic basis, mechanism, regulation, and function (Ford et al., 2006) associated with nutrient-uptake are not well understood. Root architecture is considered as a strong aspect for the improvement of NUE (Forde, 2014; Fan et al., 2017; Li et al., 2017). Broadly, root systems in cereal crops (wheat, rice, maize) can be divided into two parts a) embryonic (seminal roots), b) post-embryonic roots (crown roots). Nutrient absorption including nitrogen is well explained by “steep, cheap, and deep” root morphology (Lynch, 2013). It defines that the primary roots are involved in nitrogen acquisition from deeper horizons, whereas lateral roots with steep angles are involved in covering a greater volume of soil (Mandal et al., 2018). Lateral roots are reported to be more sensitive toward changing nitrogen content and biotic and abiotic stress. Low nitrogen content at the initial stages positively affects the lateral root initiation but severe nitrogen deficiency hampers root emergence and elongation. A high ratio of nitrate to ammonia in the soil showed a positive effect on lateral root length (Qin et al., 2017).

**FIGURE 4:**
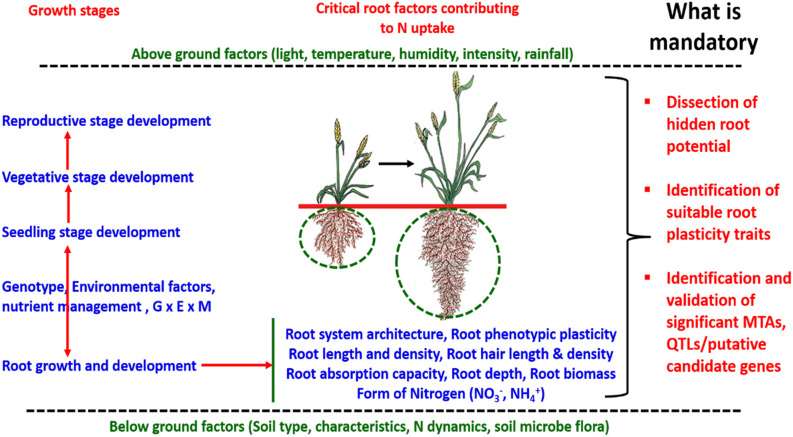
The schematic representation of the role of above and below ground factors, genotype-environment-nutrient interactions playing significant roles in developing root architecture at different stages of growth and development for efficient nitrogen uptake in cereal crops.

Addressing the challenge of efficient nutrient uptake by understanding the role of root traits in nutrient uptake and dissecting the genetic basis to maximize the potential to breed high yielding resource-efficient varieties of cereal crops using modern biotechnological and bioinformatic approaches is required. Dissecting the hidden potential of root traits for improving nutrient uptake and revealing the significant marker associations to be deployed in molecular breeding to breed resource-efficient varieties is mandatory. The exploitation of an appropriate root prototype and robust marker-trait associations/QTL/candidate genes may address the challenge of nutrient deficiency and low nutrient uptake. Efforts involving designing robust root system architecture providing a combination of different root traits (nodal root, root hair length, root hair density, root length density, root dry weight,% lateral root, root branching, root thickness, and root volume) may be a solution to the problem of efficient nutrient uptake especially nitrogen (N) (Figure 4). Various above and below ground factors are reported to play a significant role in the development of root architecture (Li et al., 2017). Different root traits are important for nutrient uptake at different stages of crop growth and development. Root size and morphological features are directly correlated with nitrogen uptake efficiency, as it is observed that among different forms of nitrogenous compounds present in soil especially nitrate easily escapes the soil system through leaching which initiates the need to enhance nitrogen uptake by improving root architecture including depth, density, and capacity of roots for post-anthesis N uptake (Foulkes et al., 2009). Primary studies to establish the molecular control of root architecture were carried out in Arabidopsis but several homologs were reported in rice and other cereal crops (Forde, 2014; Shahzad and Amtmann, 2017). Previous studies reported several genes/proteins associated with root architecture in different cereal crops. In rice, miR444a/ANR1 induces lateral root formation under low nitrate conditions (Yan et al., 2014). EL5, a plant-specific ATL Family E3 Ubiquitin ligase, maintains the viability of root apical meristem (Mochizuki et al., 2014; Nishizawa et al., 2015). OsMADS25 was reported to be involved in lateral and primary root development (Yu et al., 2015) and nitrate assimilation-related component 1 (*OsNAR2.1*) induced lateral root formation in rice (Huang and Schiefelbein, 2015). Similarly, in wheat, NAM, ATAF, and CUC transcription factor (TaNAC2-5A) promoted root growth (He et al., 2015) and the NUCLEAR FACTOR Y (TaNFYA-B1) accelerated root development (Qu et al., 2015).

A specifically in-depth role of root proliferation to increase nitrogen uptake was reported in wheat (Carvalho and Foulkes, 2011). So, the rooting profile mandate for nitrate uptake at lower depths was analyzed by measuring root length density with a threshold value of 1 cm/cm^3^ (Gregory and Brown, 1989), where root length density is a measure of root length per unit volume of soil (Ford et al., 2006; Reynolds et al., 2006). Although these threshold values for nitrogen uptake are not absolute and are highly influenced by both genetic and environmental factors. A very high genetic variability in the root system was observed in wheat (Ehdaie et al., 2003; Ford et al., 2006). Further, several environmental factors including soil type and available nutrient resource majorly affects the root distribution characteristics. *Aegilops tauschii* (D genome), the wild relative of wheat, was reported to have deeper rooting systems (Reynolds et al., 2000). The identified candidate genes may be targeted in genomics-assisted breeding programs for the development of cultivars with relatively deep rooting systems. Under low nitrogen conditions, increase in the ratio of root biomass to total plant biomass (root dry weight ratio; RDWR) was observed to maintain the functional equilibrium between the roots and shoot growth (Robinson, 2002). An intricate relation between root and shoot development in higher plants was reported, viz., active shoot growth ensures carbohydrate supply to roots to enhance the root function, whereas active root growth improves shoot growth by xylem flow of the required amount of nutrients and phytohormones to the developing shoots. The simultaneous growth of root and shoot ensured enhanced crop productivity (Yang et al., 2004; Zhang et al., 2009). The increase in root-shoot biomass even at a low nitrogen supply ultimately enhanced the crop growth rate (CGR) contributing to higher grain yield and improved NUE (Ju et al., 2015).

Along with root length and density, another important trait under consideration for enhanced nitrogen uptake is root hairs which have a substantial role in increasing the surface area of roots to potentially increase the nitrogen uptake by active transport. Among several candidate genes, two genes, i.e., RTH1 and RTH3, for root hair elongation have been identified in maize (Hochholdinger and Tuberosa, 2009). Although root structure and function seem to be an outcome of the additive effect of multiple genes so it is difficult to target single genes for amplified nitrogen uptake (Hall and Richards, 2013). The approach to enhance nitrogen uptake includes pyramiding multiple beneficial traits marker-assisted selection. The quantitative trait loci (QTL) for traits including root length, root hair number, root density, root angle, and root-to-shoot ratio are well established in wheat (Bai et al., 2013; Atkinson et al., 2015), but there is need to understand the mechanism of orchestrated expression of multiple traits affecting root architecture to positively influence nitrogen uptake (Lynch, 2007).

#### Root N Transporter Systems

Substrate specific transporters are involved in nitrogen uptake in several forms including nitrate (NO_3_^–^), ammonium (NH_4_^+^), amino acids or peptides, and urea (Crawford and Glass, 1998; Kant, 2017). Nitrogen accumulation by root is an active process mediated by a specific type of transport protein for nitrogen uptake. The inorganic form of nitrogen which is most prominent in the rhizosphere is NO_3_^–^, along with it NH_4_^+^ is also present in the soil but its concentration is significantly less compared to NO_3_^–^ concentration (Nieder et al., 2010). The uptake and transport of nitrate in plants is mediated by five transporter families including; the Nitrate Transporter 1/Peptide Transporter (NPF) family (Léran et al., 2014), the Nitrate Transporter 2 (NRT2) family, the Chloride Channel (CLC) family, the Slow Anion Associated Channel Homolog (SLC/SLAH) family, and aluminum-activated malate transporters (ALMT) (Li et al., 2017). Among the five families mentioned above NPF and NRT2 were reported to be associated with nitrate uptake and their localization in plants. The primary uptake of both NO_3_^–^ and NH_4_^+^ is mediated by diffusion or mass flow, respectively, which ensures entry of both inorganic forms to root apoplast (Mandal et al., 2018). Active transport is the prominent mechanism that further ensures the transport of nitrogenous compounds through several layers of ground tissue leading to the plant vascular system (xylem). Several types of plasma membrane-associated transporter proteins were reported to be involved in active transport and classified as high- and low-affinity transporters (Loqué and Wirén, 2004; Glass, 2009; Dechorgnat et al., 2010). Based on affinity and NO_3_^–^ concentration in the rhizosphere, three types of transport system including inducible high-affinity transport system (iHATS), constitutively expressed high-affinity transport system (cHATS), and non-saturable low-affinity transport system (LATS) are active in higher plants. iHATS is triggered at a low concentration of NO_3_^–^ (1 to 200 lM) and its functioning varies with plant species and environmental condition (Siddiqi et al., 1991; Feng et al., 2011). In wheat, iHATS has a Michaelis constant (Km) approximately 27 lM and needs an induction period of 10 h before initiating the transport process (Goyal and Huffaker, 1986). cHATS as the name suggests is constitutively expressed and displayed on the plasma membrane even in the absence of NO_3_^–^. A common property of both cHATS and iHATS is that they are saturated after the external NO_3_^–^ concentration reaches a certain threshold. The third one, LATS has low-affinity transporters and is activated at the higher concentration of NO_3_^–^ in the soil (250 lM). Unlike cHATS and iHATS, LATS includes a non-saturable type of transporters (Siddiqi et al., 1991; Von Wirén et al., 1997). Two major gene families involved in NO_3_^–^ transport in higher plants include NRT1 and NRT2. NRT1/PTR represents nitrate transporters, the peptide transporter family (NPF), and the NRT2 family known as the major facilitator superfamily (MFS) (Léran et al., 2014). The high-affinity transport system in wheat is reported to be regulated by five genes (*TaNRT 2.1*, *TaNRT 2.2*, *TaNRT 2.3*, *TaNAR 2.1*, and *TaNAR2.2*) and these transporters are activated by the plant growth hormone abscisic acid in the absence of NO_3_^–^ (Cai et al., 2007). Among the three transporter systems discussed so far, LATS is involved in NH_4_^+^ uptake and LATS belongs to NH_4_^+^ permeases in the ammonium methylammonium permeases/transporter/Rhesus (MEP/AMT/Rh) family (Wirén and Merrick, 2004). The ammonium transporters (AMTs) are considered to improve NUE by generating the AMT mutant lines and analyzing the associated phenotypic effect. In rice, twelve different AMT-associated genes were broadly classified into two subfamilies: *OsAMT1* and *OsAMT2* (Li et al., 2017; Xuan et al., 2017). Post translational events such as phosphorylation controls the activity of these transporters which keeps in check the level of ammonia accumulated in the plant system (Li et al., 2017; Xuan et al., 2017).

Along with the above discussed inorganic forms, it is important to consider the mechanism of urea uptake by the root system as it is used as synthetic fertilizer in conventional agriculture (Andrews et al., 2013; Karamos et al., 2014). It is well established that the transporter for urea uptake is expressed in roots and leaves, which can mediate efficient uptake of urea followed by its hydrolysis to use it efficiently in anabolic processes (Witte, 2011). In rice, two types of transporters for urea uptake with linear Michaelis–Menten kinetics (Wang et al., 2011) was reported. In wheat, the urea uptake is very low as compared to other inorganic N sources making it difficult to measure the kinetics of urea uptake (Criddle et al., 1988). Among the five transporters present in the plant system; one is a high-affinity transporter and the rest are low-affinity transporters. High-affinity transporters come under the category of symporter which mediates the co-transport of urea and H^+^ ions, whereas low-affinity transporters are intrinsic proteins (tonoplast intrinsic protein, TIP) working as channel proteins in a pH independent manner. The expression of high-affinity urea transporters is generally regulated by ammonia, nitrate, and urea (Reddy and Ulaganathan, 2015). However, urea is majorly used as an N fertilizer in Asian agriculture but there is a need to further investigate the mechanism of urea uptake and its metabolic conversion to useful physiological components in plant systems.

#### Effect of Rhizospheric Associations

The rhizosphere is the region of soil that comes under direct contact with the root system and the organisms surviving in this region highly influence the mineral uptake including nitrogen uptake by roots (Richardson et al., 2009). Many higher plants including wheat are reported to secrete a variety of exudates mainly organic acids and certain sugars which directly influence the physiological processes of microorganisms living in association with the root system (Nguyen, 2003). Along with this, several environmental factors including climatic conditions, water level, soil type, and farming practices also affect these microbial communities (Mazzola et al., 2004). Rhizosphere microbial ecology is also reported to be varied concerning different wheat cultivars (Kapulnik et al., 1987; Germida and Siciliano, 2001; Wu et al., 2001). There are certain cultivars possessing the efficiency to positively influence root architecture which favors N availability in the rhizosphere, systemic plant metabolism, and microbial photoprotection. Along with beneficial or symbiotic organisms, there are certain microorganisms including bacteria and fungi, which compete with the plant root system for common nitrogen sources in the rhizosphere, i.e., ammonia and nitrate (Nelson and Mele, 2006). Along with competing for the common nitrogen source, certain microorganisms negatively influence the nitrogen uptake by channeling the available inorganic nitrates to gaseous nitrogen by process of denitrification (Herold et al., 2012). In higher plants, it is also observed that certain secondary metabolites released by roots have an inhibitory effect on the denitrification process. As discussed above, denitrification converts the nitrogen into an unavailable form, so inhibition of such processes positively influences the nitrogen uptake, but such a mechanism is not well elucidated in cultivated cereal crops (Bardon et al., 2014). There were several attempts made to transfer the beneficial traits influencing root-microbial from wild relatives of cultivated cereal crops. A chromosome of *Leymus racemosus*, a wild relative of wheat possessing the ability of nitrification inhibition in the root rhizosphere, was introduced into cultivated varieties (Subbarao et al., 2007; Ortiz et al., 2008).

The nitrogen uptake by root can be improved by improving nitrogen fixation. Unlike legumes, in cereals the nitrogen fixation is not dependent on symbiotic root-nodulating bacteria, whereas this process in cereals including wheat is entailed by other non-nodulating N-fixing bacteria contributing a subtle amount of fixed nitrogen to roots in the rhizosphere (Behl et al., 2012). Although, these N-fixing bacteria form the natural component of the root rhizosphere in wheat (Nelson and Mele, 2006; Venieraki et al., 2010), but the artificial introduction of N fixers may enhance nitrogen uptake which positively influences the yield (Behl et al., 2012; Neiverth et al., 2014). Genetic engineering is the major solution that can introduce the legume-like system of nitrogen fixation from bacteria to the cereal crops (Geddes et al., 2015). Previous studies reported several strains of bacteria that can be used as cereal seed inoculants to naturally fix nitrogen or can act as potential hosts to receive the gene clusters for nitrogen fixation. The most potent strains that can be targeted for nitrogen fixation are non-host-specific endophyte Pseudomonas stutzeri and epiphyte Klebsiella oxytoca known to colonize with the root system of rice and wheat (Triplett et al., 2008). Bacterial systems carry a diverse range of *nif* gene clusters ranging from 11 kb to 64 kb operons. The conserved region among these operons includes nitrogenase (nifHDK) and cofactor (FeMoCo) (Boyd et al., 2015) and the rest of the region in the operon specifies nitrogen fixation under different environmental conditions (Pascuan et al., 2015; Poudel et al., 2018). Ryu et al. (2020) compared diverse species, natural *nif* clusters, and engineering strategies to design bacteria capable of delivering fixed nitrogen to the cereal crop. Rhizosphere-associated increase in NUE is dependent on nitrogen-fixing microbial associations in cereals (Mus et al., 2016). Although there is less evidence on the occurrence of efficient diazotrophic associations in cereal crops (Van Deynze et al., 2018). Certain examples of fixed atmospheric N_2_ being transferred to cereals include associations between Azoarcus sp. strain BH72 and Kallar grass (Hurek et al., 2002), Herbaspirillum seropedicae and rice (Gyaneshwar et al., 2002), and Klebsiella pneumoniae and wheat (Iniguez et al., 2004). Several mechanisms controlled by microorganisms in the rhizosphere affecting the root architecture by increasing production of growth hormones including auxins (Ortíz-Castro et al., 2009), cytokinins (Cassan et al., 2009; Moubayidin et al., 2009) or gibberellins were detected. The gibberellins secreted by several bacteria and fungi (Bottini et al., 2004) in the rhizosphere enhanced the primary root elongation and lateral root extension in wheat (Upadhyay et al., 2009). The root-associated organisms produced a vast effect not only on nitrogen uptake but also on triggering plant defense systems against pathogenic organisms (Couillerot et al., 2011; Almario et al., 2013). In wheat, the pathogenic defense-related transcriptional accumulates increased in wheat when inoculated with the bacterium *Pseudomonas fluorescens* Q8r1-96 (Maketon et al., 2012). Overall, the microbial association in respect to nitrogen uptake is a broad subject that needs to be considered and explored for further improvement of nitrogen uptake efficiency in wheat and other cereal crops.

### Traits Affecting Nitrogen Utilization Efficiency

#### Nitrate Assimilation

**FIGURE 5:**
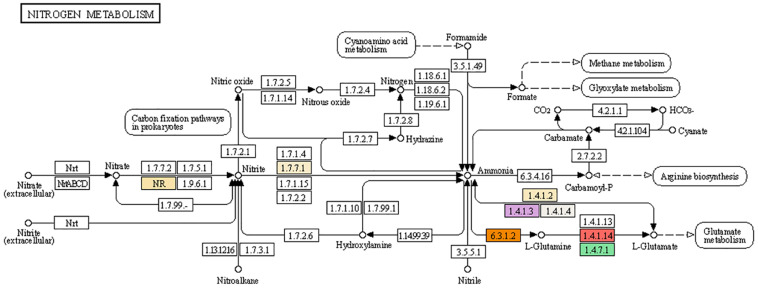
Mechanism of nitrogen assimilation and integrated pathways with different enzymes involved in channelizing nitrate toward amino acids and proteins. Nitrate and nitrite are the primary nitrogen source for cereal crops. Nitrate is converted to nitrite by regulatory enzyme: nitrate reductase (NR, highlighted in creamish color) (1.7.7.2). Nitrite is further reduced to ammonia by nitrite reductase (1.7.7.1, highlighted in creamish color). Ammonia is channelized for amino acid synthesis primarily by action of glutamate dehydrogenase (1.4.1.2, highlighted in creamish color¶and 1.4.1.3, highlighted in purple color). Glutamate is further converted to glutamine by glutamine synthetase (6.3.1.2, highlighted in orange color; 1.4.7.1, highlighted in green color and 1.4.1.14, highlighted in red color). Glutamine and glutamate are the primary amino acids routed for protein synthesis. Partial fraction of ammonia is involved in arginine metabolism using carbamoyl phosphate as C-skeleton. In addition to the primary nitrogen metabolism, secondary nitrogen sources such as nitric oxide, nitrous oxide, nitroalkane, nitrile, hydrazine, and formamide also contribute in nitrogen metabolism. Cyanoamino acid metabolism releases formamide which converts to formate for further used in methane and glyoxylate metabolism.

In higher plants, the major pathway for inorganic nitrogen assimilation into the carbon skeleton is nitrate reduction (Ali, 2020). The mechanism of nitrate assimilation involves reduction and its conversion into biologically active forms as described in detail in Figure 5. Nitrate, the primary form of nitrogen taken up by the roots, is reduced to nitrite by NAD(P)H-dependent enzyme nitrate reductase (NR) inside the cytoplasm (NR; EC 1.7.1.1). Nitrate reductase exists in homodimeric forms with subunits of about 900 amino acids (110kDa). Each subunit is associated with FAD, heme-Fe, and Mo-molybdopterin (Mo-MPT). Nitrate reductase along with a molybdenum cofactor (MoCo) is needed to catalyze the rate limiting step of nitrate reduction. As it is the major rate limiting step, it is highly regulated at the gene expression level by several factors (Campbell, 2002). NR activation is the very first step in the utilization of absorbed nitrogen for its conversion into biologically active molecules including amino acids, nucleic acid, and other nitrogen-containing biomolecules. Two genes encoding NADH-dependent nitrate reductase was reported in hexaploid wheat (Boisson et al., 2005).

The second step catalyzed by nitrite reductase reduces the nitrate to ammonia (NO_2_^–^ to NH_4_^+^) and this enzyme is compartmentalized in the plastids (NiR; EC 1.7.7.1; Sétif et al., 2009). The electron donor for the reduction of nitrite into ammonia is provided by ferredoxin by forming an enzyme-ferredoxin complex (Sakakibara et al., 2012). The incorporation of ammonium into the carbon skeleton is mediated by 2-oxoglutarate of the TCA cycle and amino acid transamination reactions to form glutamate and glutamine. Ammonia forms after two subsequent reactions are incorporated into an organic form. Glutamate is the amino acid that acts as a primary receiver of ammonia and this reaction is catalyzed by consecutive action of the two enzymes. Glutamine synthetase catalyzes the first reaction (GS; EC 6.3.1.2; Lea and Miflin, 2011) and is a major regulatory step in channeling the inorganic form of nitrogen to its organic form.

GS has two isoforms and works in different cellular compartments, the first isoform (GS1) is mainly expressed in the cytosol of several organs such as leaves, roots, and phloem cells, whereas the second isoform (GS2) is expressed in plastids of chloroplast, roots, and etiolated tissues (Lea and Miflin, 2011). In cereals including wheat the GS2 isoform is expressed majorly throughout the plant development cycle and its activity decreases post-anthesis, whereas cytosolic isoform GS1 isoenzymes show constitutive expression in the phloem and senescing leaves (Christiansen and Gregersen, 2014; Yamaya and Kusano, 2014). Another enzyme glutamate synthase (GOGAT; EC 1.4.7.1; Suzuki and Knaff, 2005) acts in conjunction with the primary enzyme and catalyzes glutamate synthesis (GS) by incorporating carbon skeletons in the 2-oxoglutarate form into the cycle. Further, these two amino acids act as principal donors of amino groups for the formation of other amino acids, nucleic acids, and other nitrogen-containing compounds (Lea and Miflin, 2011). GOGAT also exists in two isoforms, each with a role in N assimilation or N recycling. One isoform of GOGAT is ferredoxin-dependent isoenzyme (Fd-GOGAT) in reassimilation of photorespiratory ammonia, whereas the other isoenzyme of GOGAT is NADH dependent (NADH-GOGAT; EC 1.4.1.14) with its role in the synthesis of amino acids including glutamate for growth and development in photosynthetic and non-photosynthetic organs (Lea and Miflin, 2011). It is described from mutational studies that GS and GOGAT contribute to the assimilation of about 95% of the ammonia available in plant tissues (Lea and Miflin, 2011). The amino acid formed is utilized in protein formation and production of other metabolites important for growth and development and to ultimately increase productivity (Howarth et al., 2008).

Nitrogen assimilation and utilization is affected by several factors but carbon metabolism is the major player affecting NUtE. The effect of photosynthesis and carbon metabolism on nitrogen accumulation was studied in different plants to analyze the role of various carbon metabolites in nitrogen utilization. The change in photosynthetic rate was reported to affect nitrogen assimilation and vice versa. Carbon fixation depends on nitrogen assimilation, as it is important to provide enzymes and pigments for photosynthesis (Kant et al., 2012) that makes nitrogen an important component describing the photosynthetic activity and crop yield. Similarly, nitrogen assimilation depends on carbon metabolism which provides the electron donors for ammonium formation, the carbon skeleton (ketoglutarate) for ammonia assimilation in GS/GOGAT pathway, malate as a counter anion, carbon precursors for other amino acid pathways, and ATP for nitrate transport into the cell (Xu et al., 2012). Therefore, several factors were reported to regulate the nitrogen assimilation and utilization process which must be considered while describing NUE (Ali, 2020). Nitrogen use efficiency is related to nitrate acquisition which can further be enhanced by altering enzymes and proteins associated with nitrate assimilation utilizing different biotechnological approaches. There is strong need to target multiple mechanisms/enzymes/factors rather than approaching single-point rate-limiting regulation to enhance NUE. Therefore, future research is dependent on in-depth understanding of the regulatory mechanisms of N metabolism and the pathways linking C and N assimilation (Ali, 2020).

#### Canopy Architecture and Photosynthesis

Photosynthesis is an important physiological process occurring in higher plants. The most abundant protein in the biosphere is Rubisco. Rubisco is a major regulatory enzyme for the process of converting the inorganic form of energy to the organic form through the Calvin cycle. As Rubisco is a protein biomolecule, most of the nitrogen accumulated in the leaf is represented by the Rubisco content which in return represents the photosynthetic activity of mesophyll tissues. It is reported in wheat that approximately 75% of N in leaves is channeled for photosynthetic processes mediated by the Rubisco enzyme (Evans, 1983). So, it is observed that in nitrogen-limited conditions there is a reduction in Rubisco content which decreases the photosynthetic activity per unit area of leaf and ultimately decreases the organic matter production. Photosynthetic activity is correlated to leaf morphogenesis as it is the primary organ involved in carbon dioxide fixation, so leaf architecture directly affects yield in crop plants (Guo et al., 2019). It is reported earlier that leaf width affects grain- and panicle-related traits (Fu, 2019). A study on rice crop suggested that leaves were the major photosynthetic organ for plant morphological development, and spatial arrangement of leaves was reported to be strongly correlated to rice yield (Zhang et al., 2020). The reduced photosynthetic activity leads changes in canopy structure including reduced leaf expansion and decreased total leaf area (Sylvester-Bradley et al., 1990; Monneveux et al., 2005). The nitrogen uptake and utilization enhanced the source and sink capacities, thereby increasing the dry matter accumulation and ultimately improving the crop yield. Traits such as spike shape, plant height, and biomass accumulation in rice were reported to be associated with nitrogen uptake and utilization (Xu et al., 2020).

Grain yield in cereals is the outcome of coordinated regulation of multiple factors including photosynthesis, nitrogen uptake, and photorespiration (Sinclair et al., 2019). The correlation between yield and the absorption, uptake and utilization of nutrients played significant roles in improving rice yields. The complete understanding of the regulatory mechanisms and pathways involved in the transport of nutrients to the stems, sheaths, and leaves and then finally to the grains and how to improve the slow and ineffective filling of grains is mandatory (Li and Cui, 2014). It is reported that LWS5/D1-mediated GA signaling regulates the GPCR (G-protein coupled receptors) in rice (Miyako et al., 2000), ultimately improving nitrogen uptake, grain yield, and regulating leaf morphology (Zhu et al., 2020).

The optimization theory indicated that the equal coefficient of the light gradient (KL) and nitrogen (KN) positively increased canopy photosynthesis (Moreau et al., 2012). Further, the nitrogen gradient reported in wheat was less steep than in the optimization theory (Moreau et al., 2012). NUtE was affected by the photosynthesis rate per unit of nitrogen. In C_3_ crops such as wheat around 2 g N/m^2^ of the leaf N concentration increased the photosynthesis to 20–30 lmol CO_2_/m^2^/s in the light-saturated condition.

Exploiting the tendency of wheat cultivars to accumulate 2.0 g N/m^2^ under the favorable conditions may be another important aspect to increase NUtE. Genetic variability in specific leaf nitrogen (SLN, leaf nitrogen content per unit leaf area) was observed to be varied from 1.4 to 2.6 g/m^2^ for the 144 durum wheat genotypes (Araus et al., 1997), from 2.1 to 2.4 g/m^2^ for the 17 durum wheat cultivars (Giunta et al., 2002), and from 1.4 to 2.2 g/m^2^ for the 16 bread wheat cultivars (Moreau et al., 2012). The heritability for straw nitrogen including stem, leaf lamina, and leaf sheath at anthesis for winter wheat was > 0.60 under low nitrogen (Laperche et al., 2006), indicating the possibility for selection for this trait.

Photorespiration catalyzed by Rubisco (ribulose-1,5-bisphosphate carboxylase/oxygenase) activates the fixation of oxygen and release of previously fixed CO_2_ and NH_3_ at the cost of energy consumption. Consequently, the fixed nitrogen is lost from the metabolic cycle. Several components can be targeted to increase the photosynthetic activity by decreasing photorespiration through Rubisco, increasing carboxylase activity of Rubisco and by introducing mechanisms to increase carbon concentration in the vicinity of Rubisco. Increased photosynthetic activity will ultimately increase NUtE (Reynolds et al., 2000; Long et al., 2006; Murchie et al., 2008; Zhu et al., 2010). Genetic diversity can be exploited to strategize the required modification in photosynthetic components. The 30% improvement in photosynthetic activity was mediated through reduced photorespiration whereas other mechanisms hold the potential of 15–22% enhancement of photosynthetic activities (Long et al., 2006). Further study is required to better understand the molecular processes regulating the signaling pathways for leaf architecture, photosynthesis, and photorespiration. Gene editing technologies such as CRISPR-Cas9 or the expression of specific promoters can be used to alter the genes regulating signaling pathways in leaves, resulting in diverse germplasms with high yield potential (Zhu et al., 2020). Precision breeding techniques are required to improve the breeding efficiency among cereals for traits enhancing NUE.

#### Post-anthesis N Remobilization and Senescence Dynamics

Nitrogen uptake from roots is further mediated by its translocation from roots to leaves through the transpiration stream where roots act as source and growing tissues such as leaves and buds act as a sink. Although this source-sink relation changes with the developmental stage, during maturity the mature leaf acts as source, where proteins are degraded and release nitrogen which is remobilized to younger leaves and seeds (Lemaire et al., 2007).

Before discussing nitrogen remobilization, it is important to consider that plants are a better option for nitrogen storage as compared to soil. In soil, nitrogen is readily converted into a different reduced and oxidized form along with processes such as denitrification which decreases the availability of the biologically active form of nitrogen. Therefore, it is important to have crop plants which can efficiently store nitrogen in different tissues and maintain it in the biological system through accumulation in grains by remobilization (Hofman and Van Cleemput, 2004).

Nitrate remobilization from leaves to developing parts is a valuable determinant of NUE during the grain-filling stage. The crop yield depends on the remobilized nitrogen. Photosynthates stored in the old leaves act as a major source of recycled nitrogen for remobilization to developing seeds. Remobilization is dependent on the mechanism of autophagy which is basically regulated by several senescence-associated genes (ATG and metacaspases) that get induced during plant senescence (Havé et al., 2016). Nitrogen replenishment during the reproductive stage is mediated by tissue-specific transporters. The genes which code for nitrogen transporters such as *AtNRT1.7* are further controlled by nitrogen limitation adaptation (NLA) regulators which are further controlled by miRNA827 (Liu W. et al., 2016). The strict control of nitrogen transporter expression suggests tight regulation of the remobilization process. Along with transporters, the enzymes such as ferredoxin-dependent glutamate synthase (OsFd-GOGAT) involved in ammonia recycling played a significant role in the remobilization process (Zeng et al., 2016).

In cereal crops, approximately 50–90% of nitrogen accumulated in grains were contributed to by remobilized nitrogen from leaves (Masclaux-Daubresse et al., 2010). Flag leaf senescence can be used as a phenotypic marker to estimate the stage of nitrogen remobilization to grains (Uauy et al., 2006). Although, delayed senescence of flag leaf led to higher grain yield, which persists with the results suggesting an inverse relationship between grain yield and grain protein content. As Rubisco is the most abundant protein present in the chloroplast of photosynthetically active tissues, i.e., leaves, during remobilization Rubisco is a major contributor of nitrogen for protein accumulation in grains. It is suggested that older leaf tissue chloroplasts degrade first in comparison to other organelles due to upregulation of proteases. Autophagy is the underlying mechanism for chloroplast and Rubisco degradation during senescence (Ishida et al., 2014; Li F. et al., 2015). The process of autophagy is mediated by exopeptidases and endopeptidases present in cell vacuoles (Ishida et al., 2014). The amino acids released after degrading the Rubisco protein transported via amino acid transporters belonged to the amino acid permeases (AAP) family (AAP1, AAP2, AAP3, AAP6, AAP7, AAP8 and AAP16) (Hunt et al., 2010; Taylor et al., 2015).

In wheat, nitrogen content in the above-ground tissue during anthesis is maximally in leaf lamina followed by the true stem, ear, and leaf sheath under optimal N supply (Barraclough et al., 2014; Gaju et al., 2014). However, under limiting nitrogen conditions the nitrogen content in the ear increases in comparison to other plant parts (Barraclough et al., 2014; Gaju et al., 2014).

The efficiency of post-anthesis nitrogen remobilization of true stem reserve N was low (48%) compared to the leaf sheath (61%) and leaf lamina (76%), but true stem acted as a major reservoir of nitrogen during harvest in well-fertilized crops. Theoretically, before anthesis true stem has a high capacity to store nitrogen which enhances nitrogen uptake and favors high NUpE (Foulkes et al., 2009). This condition of high nitrogen in stem (non-photosynthetic tissue) further facilitates the nitrogen translocation for grain filling without hampering photosynthetic capacity (Bertheloot et al., 2008), but to ensure benefits of storing nitrogen in non-photosynthetic tissue it is necessary to study the respiratory cost associated with it. Huge diversity among wheat germplasm for nitrogen storage and remobilization in non-photosynthetic tissues especially in stem during anthesis was reported (Kichey et al., 2007; Barraclough et al., 2014; Gaju et al., 2014). The shoot not only acts as the non-photosynthetic storage tissue for nitrogen but it also has regulatory control over N uptake from roots, and allocation to sink. The high accumulation of amino acid in phloem tissue positively affects nitrogen uptake from root followed by its assimilation in storage tissue (Zhang et al., 2015; Perchlik and Tegeder, 2017). Proper nitrogen partitioning in various tissues including shoot was reported to be important for C/N metabolism (Santiago and Tegeder, 2017).

Genetic diversity in terms of senescence and ‘stay-green’ phenotypes was reported in hexaploid wheat (Bogard et al., 2011; Gaju et al., 2011; Derkx et al., 2012; Naruoka et al., 2012). The stay-green phenotype acts as a mark for the capacity of a germplasm to stay green during the grain filling stage, i.e., retains its photosynthetic activity in comparison to other genotypes under consideration (Thomas and Smart, 1993). These factors including Rubisco degradation, stem nitrogen assimilation, and stay-green phenotypes provide major targets to ensure active remobilization of nitrogen to the grains post-anthesis. The molecular studies reported certain transcription factors such as NAM-B1 which efficiently increases nitrogen remobilization in wheat by accelerating the senescence of leaves (Uauy et al., 2006). The members of the WRKY and NAC transcription factors family acted as regulatory genes through their role in senescence under controlled environment conditions (Derkx et al., 2012). There was an association reported between QTL affecting leaf senescence, grain yield, grain protein content, and QTL for the anthesis period in a double-haploid mapping population of winter wheat. The post-anthesis nitrogen availability for grain filling depends upon leaf senescence and flowering time (Bogard et al., 2011). Grain yield and storage protein synthesis was reported to be highly correlated to nitrogen (N) uptake after anthesis and remobilization of nitrogen from pre-anthesis uptake synthesis (Dupont and Altenbach, 2003). Kichey et al. (2007) reported that a less significant fraction of grain storage protein was synthesized from post-anthesis nitrogen uptake. The aim of achieving both high grain yield and protein content in wheat depends on better understanding of the mechanisms behind post-anthesis nitrogen remobilization which can be exploited to achieve the desired outcomes under variable environmental conditions.

#### Grain Protein Content and Grain Yield

The endosperm is the major edible part of cereal grains and its nutritional value is defined by the composition of metabolic products accumulating in mature grain. Although, starch is the major constituent of the cereal endosperm, protein is also present in association with starch. Gluten storage protein forms the major fraction of protein accumulated in the endosperm, which is composed of an admixture of polymeric glutenins and monomeric gliadins. Overall, these storage proteins are from the 60–70% nitrogen store of seed endosperm. In wheat, the dough-making property essential for forming multiple consumable products including bread, pasta, and noodles is ensured by gluten protein and there is a need for the precise balance between the ratios of gliadin which ensure dough viscosity and glutenins responsible for dough elasticity. However, grain protein quality varies under different genetic backgrounds which affect the capacity of protein synthesis and nitrogen utilization (NUtE) (Shewry and Halford, 2002; Ravel et al., 2009). Environmental factors also affect several components of grain development including rate, duration of grain filling, and grain protein composition (Martre et al., 2003). Grain yield and grain protein content are two important components affecting NUtE but they are inversely related to each other (Oury and Godin, 2007; Bogard et al., 2010) which creates an obstacle in achieving both simultaneously. The metabolic competition between carbon and N fluxes for energy leads to the physiological basis of the inverse relation between grain yield and grain protein content (Munier-Jolain and Salon, 2005), so dilution in NUtE is in the effect of accumulated carbon-based compounds (Acreche and Slafer, 2009). The efficient nitrogen retained in grain can be calculated by considering grain protein deviation (GPD), which gives a measure of deviation from the regression between grain protein concentration (GPC) and grain yield. Identification of genotypes with higher GPC from an expected GY can be estimated by calculating GPD (Monaghan et al., 2001). Post-anthesis N uptake is mainly affected by genetic variability in cereal (Monaghan et al., 2001; Bogard et al., 2010, 2011). Along with it, another major consideration is that after anthesis the main source of nitrogen for grain is remobilization from other metabolically active tissues, rather than nitrogen uptake from the rhizosphere (Gaju et al., 2014), so there is a need to address the remobilization and efficient storage of nitrogen in photosynthetically active tissues to increase the GPD (Hawkesford, 2014). The increase of 16.6% grain yield in rice was reported with increasing nitrogen supply due to an increase in the productive tiller number (Liu X. et al., 2016). Similarly, yield improvement was observed in barley due to the improvement in the yield attributing components such as the number of productive tillers, grain size, and number of grains spike^–1^ (Beatty et al., 2010; Safina, 2010; Ghoneim et al., 2018).

## Interactive Nitrogen Metabolism

Nutrient enrichment in plants depends upon interactive uptake, storage, mobilization, and translocation of micronutrients and macronutrients including nitrogen. These complex processes are regulated by coordinated interaction of multiple genes (Jin et al., 2013). Ionomic studies revealed variation in a given subset of elements in the rhizosphere leading to change in the macro- and micro-nutrient status of plants (Murgia and Vigani, 2015). Further, the nitrogen source in the rhizosphere affected the micro and macro-nutrient profile ultimately affecting core metabolic processes such as photosynthetic rate, NUE, growth, and yield (Na et al., 2014). Therefore, nitrogen use efficiency can be enhanced by synchronized increase in mineral uptake along with nitrogen. In a superficial view it was observed that decline in nitrogen content subsequently decreases uptake and utilization of other mineral nutrients including P, K, Mg, Ca, Cu, Fe, and Mn (Waters and Sankaran, 2011). Although nitrogen metabolism and its interaction with other nutrients varies with respect to environment, genotype, tissue, and nutrient. The K and P content in the roots were observed to be affected with varying nitrogen level as compared to Ca and Mg, whereas variation in K content was much smaller than Mg content in shoot (Shah et al., 2017). The low nutrient level was reported to elicit the expression of transporter proteins for coordinated uptake of nutrients such as nitrogen, phosphorous, and sulfur (Gojon et al., 2009). The synergetic effect of nitrogen fertilizer application led to cumulative uptake of nutrients such as P, K, Ca, Fe, Cu, and Mn in both leaves and roots (Shah et al., 2017).

As discussed in nitrate assimilation, nitrate reductase (NR) is essential for nitrate to nitrite conversion. NR activity is dependent on the presence of molybdenum cofactor (Moco) along with the availability of nitrite ions, growth condition, phosphorylation, and hormonal induction (Garg, 2013; Nemie-Feyissa et al., 2013). Molybdenum plays an essential role in nitrogen assimilation as it is a component of Mo-cofactor which is important for enzymes involved in plant growth and developmental processes. Mo act as cofactors for glutamate synthase enzymes involved in ammonia incorporation into amino acids (Liu et al., 2017). The deficiency of Mo led to poor nitrogen assimilation and plants showed symptom of nitrogen deficiencies indicating the key role of Mo in nitrogen metabolism (Kaiser et al., 2005). Mo was reported to significantly affect activities and expression of NR and other enzymes involved in nitrate assimilation (NiR, GS, GOGAT enzymes) (Imran et al., 2019). Remobilization from the older tissue was reported as a secondary mechanism to combat the nitrogen demand in case the uptake mechanism failed (Etienne et al., 2018). As most of the macro and micronutrients form part of the complex proteins including enzymes and pigments, degradation of these complex proteins channelizes the remobilization of nitrogen along with elements such as Zn, Cu, Mn, and Fe (Ono et al., 2013). A chromosomal locus in wild wheat was reported to regulate the early senescence and remobilization of protein associated with higher mobilization of N, Zn, Mn, and Fe from leaves to the seeds. Remobilization of Cu, Zn, and Fe was reported to be tightly linked with N catabolism during senescence (Waters and Sankaran, 2011). Similarly, delayed leaf senescence was observed to be associated with lower amounts of Fe and Zn in wheat seeds (Uauay et al., 2006). Nitrogen and other related nutrients (P, K, Ca, S, Mg, Fe, Zn, Cu, Mn) were reported to be negatively influenced by increased seed carbon concentration (Loladze, 2014). The seeds and leaves were reported to store large starch granules in chloroplasts under nitrogen-deficient conditions (Bhaskar and Syvertsen, 2005). Overall, the processes of macro and micronutrient assimilation, storage, and mobilization are interlinked, which provide multiple targets to enhance the NUE of cereal crops.

## Biological Nitrification Inhibition

Nitrification is a key process, mediated by soil microorganisms, which converts reduced nitrogen (N) from ammonium (NH_4_^+^)/ammonia (NH_3_) (an immobile form of N) to nitrate (NO_3_^–^) (a mobile form of N) via nitrite (NO_2_^–^). The mobile nature of the nitrification product NO_3_^–^ leads to the loss of N in the form of leaching causing groundwater pollution and leads to gaseous N_2_ via denitrification causing air pollution. Also, the nitrification process leads to the acidification of the soil facilitating the leaching of other important cations, i.e., Ca^2+^, Mg^2+^, and K^+^. The suppression of nitrification and denitrification minimizing the loss of ammonium fertilizer post-application are very critical steps to improve the retention of N fertilizer in soils, thus improving the N-use efficiency (NUE) of cereal crops with a view toward agricultural production and environmental sustainability. The use of synthetic inhibitors such as dicyandiamide (DCD), 3,4-dimethyl pyrazole phosphate (DMPP), 2-(N-3,4-dimethyl-1H-pyrazol-1-yl) succinic acid isomeric mixture (DMPSA), and nitrapyrin to reduce nitrification has been restricted because of the inconvenience of application, lack of availability, high cost, and their potential for environmental contamination. Considering these constraints, it is very much necessary to develop plant-derived environmentally friendly nitrification inhibitors to suppress soil-nitrifier activity which are referred to either as natural nitrification inhibitors (NNIs) or biological nitrification inhibitors (BNIs) (O’Sullivan et al., 2016). Recently, new methods have been developed to study soil N transformations to significantly reduce nitrification through root exudation. These compounds reportedly blocked the ammonia-monooxygenase and hydroxylamine-oxidoreductase enzymatic pathways in the soil microorganisms responsible for the oxidation of NH_4_^+^ to NO_2_^–^.

## Genetic Factors Involved in Nue

### QTL Related to NUE

NUE is a quantitative trait controlled by multiple genes (Yang et al., 2017). QTL mapping is a powerful tool to dissect the complexity of quantitative traits (Sun et al., 2012). Successful QTL mapping for complex traits including NUE is dependent on various factors such as the selection of suitable parents, appropriate population size, multi-location testing, and genome coverage. QTL can be affected by environmental variation. Constitutive QTL is consistent over environments, while adaptive QTL shows expression in a specific environment, or modulates their effect with changing environmental conditions. QTL analysis provides opportunities to identify the relationships between different traits. Co-localization of the QTL linked with different phenotypically correlated traits is good evidence that the traits might be genetically and functionally linked.

The NUE of cereal crops can be improved by employing classical genetics that involve both conventional breeding and QTL mapping in combination with MAS (marker-assisted selection). With the identification of agronomically relevant traits and the advances of next-generation sequencing, it is feasible to develop genomic knowledge for cereal crops even with complex genomes such as wheat (Guo et al., 2011). Identification of cheap, easy to use, widely distributed, co-dominant, trait-associated, and regulatory SNPs, candidate genes, and regulatory pathways could represent a significant milestone to accelerate the global hunt to improve wheat. Identification of genes with non-synonymous and regulatory SNPs could functionally differentiate accessions based on their distinct agronomic traits. Crop improvement programs can use association studies to access allelic diversity and to identify the best alleles to be assembled in superior varieties. The utilization of high-throughput genotyping techniques has the potential to increase marker density and may thus improve the accuracy of the identified QTL for nitrogen uptake and utilization-related traits. Several promising ways to improve NUE have been proposed such as focusing on root architecture (Foulkes et al., 2009) or senescence and remobilization (Gaju et al., 2011; Distelfeld et al., 2014). The ability to identify major and stable QTL controlling NUE-related traits and the use of this available information and knowledge in crop improvement breeding programs may condition part of the future cereal crop genetic gain.

Previous studies reported various QTL for NUE in the model crop plant, i.e., Arabidopsis and also in other cereals such as maize, rice, and wheat (Agrama et al., 1999; Gallais and Hirel, 2004; Ribaut et al., 2007; Li et al., 2017). Cho Y. I. et al. (2007) reported QTL for grain and shoot nitrogen content, harvest index, and physiological NUE under both low and the normal N on rice chromosomes 5, 7, 8, 9, and 10 using RILs. Similarly, Wei et al. (2012) identified QTL for nitrogen response, grain yield, and physiological NUE in rice. Further, significant QTL for grain yield, root NUE, and shoot dry weight have been detected in the wheat RIL population, i.e., TN18 × LM6 (Zhang et al., 2019). A total of 13 QTL including 7 QTL for nitrogen uptake and 6 for NUE were identified in rice grown under hydroponic culture (Zhou et al., 2017). The proportion of total phenotypic variation explained by QTL for NUP ranged from 3.16 to 13.99% and NUE QTL ranged from 3.76 to 12.34%. A major QTL on the short arm of chromosome 6B controlling grain protein content in wheat accounting for 66% of the phenotypic variation was reported and the functional gene named *Gpc*- *B1* was cloned (Uauy et al., 2006). In winter wheat, the QTL associated with NUE on chr 1D, 6A, 7A, and 7D with LOD scores ranging from 2.63 to 8.33 and phenotypic variation up to 18.1% were identified (Brasier et al., 2020). Various novel NUE-related traits and alleles in adapted breeding materials (Fontaine et al., 2009), landraces (Pozzo et al., 2018; Van Deynze et al., 2018), and wheat wild relatives (Hu et al., 2015) were identified. QTL associated with NUE in rice were mapped using a recombinant inbred line (RIL) population on chromosome 6 (qNUEP-6; Shan et al., 2005) and on chromosome 9 (pnue9; Cho Y. I. et al., 2007). Thus, the research work carried out on cereal crops such as rice, maize, and wheat set a precedence for NUE research in other cereal crops such as barley (Cho Y. et al., 2007; Xu et al., 2014; Li P. et al., 2015; Lei et al., 2018; Mandolino et al., 2018).

Cormier et al. (2016) proposed that the identification of the genomic regions (QTL) associated with nitrogen response would enable more efficient cultivar selection. This approach allows breeders to efficiently screen germplasm and the genetic markers associated with nitrogen response, assisting in the development of high nitrogen use efficient cultivars. Previous studies have been conducted in rice and wheat to identify the novel traits, alleles, genes/QTL, adapted breeding lines, landraces, and wild relatives improving NUE differences in cereal crops. Genes/QTL influencing nitrogen uptake have been mapped in wheat under different doses of fertilizer application using bi-parental populations (An et al., 2006; Laperche et al., 2007b; Xu et al., 2013; Mahjourimajd et al., 2016). QTL for nutrient uptake was reported to be collocated with QTL for root hair length (Sandhu et al., 2015) and grain yield with root architectural plasticity traits (Sandhu et al., 2016) in rice. Several genetic loci for agronomic traits related to nitrogen use and grain yield have been mapped in the chromosomal regions containing GS2 in wheat and rice (Prasad et al., 1999; Obara et al., 2001; Yamaya et al., 2002; Habash et al., 2007; Laperche et al., 2007b; Fontaine et al., 2009; Yamaya, 2011), suggesting the role of the genomic region surrounding GS2 in breeding wheat and rice varieties with improved agronomic performance and nutrient use efficiency. Detailed descriptions of the QTL associated with nitrogen use efficiency-related traits and nitrogen use efficiency in rice are presented in Tables 1, 2, respectively and in wheat are presented in Tables 3, 4, respectively.

**TABLE 1:** Detailed description of QTL associated with nitrogen use efficiency-related traits in rice.

Traits	QTL name	Chr	Marker/interval(bp)	LOD/*F* value	ADD	*R*^2^	References
100 grain weight	*qHGW-1a*	1	MRG0195-RM490	4.1	0.04	2.4	Tong et al., 2010
	*qHGW-3*	3	RM282-MRG0164	13.9	0.09	9.8	Tong et al., 2010
	*qHGW-5a*	5	RM405-RM574	7.8	0.05	2.7	Tong et al., 2010
	*qHGW-6*	6	RM564-RM541	8.1	−0.05	2.7	Tong et al., 2010
	*qHGW-10b*	10	RM147-RM228	7.4	0.06	4	Tong et al., 2010
	*qHGW-1a*	1	MRG0195-RM490	6.1	0.06	3.3	Tong et al., 2010
	*qHGW-3*	3	RM282-MRG0164	15	0.1	10.4	Tong et al., 2010
	*qHGW-5a*	5	RM405-RM574	5.1	0.04	1.4	Tong et al., 2010
	*qHGW-6*	6	RM564-RM541	5.3	−0.04	1.6	Tong et al., 2010
	*qHGW-10a*	10	MRG4392-RG5477	6.7	0.04	1.3	Tong et al., 2010
	*qHGW-10b*	10	RM147-RM228	4.5	0.05	2.3	Tong et al., 2010
	*qHGW-1b*	1	RM490-RM243	11.9	0.05	2.5	Tong et al., 2010
	*qHGW-2*	2	RM3355-RM263	7.5	−0.04	1.4	Tong et al., 2010
	*qHGW-5a*	5	RM405-RM574	9.8	0.04	1.9	Tong et al., 2010
	*qHGW-5b*	5	MRG2870-RM274	4.2	−0.03	1.2	Tong et al., 2010
Biomass yield	*qRBM9-1*	9	RG570-RM242	2.85	−0.02	8.4	Wei et al., 2012
	*qRBM9-2*	9	RM242-RM257	4.47	−0.02	13.1	Wei et al., 2012
	*qRBM10*	10	C909a-R2174	2.75	0.01	12.2	Wei et al., 2012
	*qRBM1*	1	RG101-C922	3.01	−0.02	11.5	Wei et al., 2012
	*qRBM2*	2	C777-R1843	3.06	−0.02	9.3	Wei et al., 2012
	*qRBM4*	4	G235-G102	3.84	0.03	14.5	Wei et al., 2012
	*qBY1.1*	1	40660285–40695764	19.22	–	11.47	Zhou et al., 2017
	*qBY2.1*	2	36017977–36777825	7.18	–	3.21	Zhou et al., 2017
	*qBY2.2*	2	36777825–36823111	9.55	–	3.98	Zhou et al., 2017
	*qBY3.1*	3	12844058–13297480	9.19	–	45.54	Zhou et al., 2017
	*qBY6.1*	6	7814673–9668398	8.71	–	4.6	Zhou et al., 2017
	*qBY8.1*	8	2797908–3336084	22.05	–	15.1	Zhou et al., 2017
	*qBY10.1*	10	22335288–22517954	8.93	–	5.01	Zhou et al., 2017
	*qBY11.1*	11	25559185–26317711	7.1	–	3.34	Zhou et al., 2017
Chlorophyll content index	*qCCL*	3	RM416–RM293	4.49	1.585	12.4	Nguyen et al., 2016
Dry weight of blades	*qDWB*	3	RM293–RM468	3.93	0.073	11.38	Nguyen et al., 2016
Dry weight of roots	*qDWR*	3	RM293–RM468	5.2	0.036	14.44	Nguyen et al., 2016
	*qDWR*	8	RM042–RM284	3.58	−0.031	9.74	Nguyen et al., 2016
Dry weight of shoots	*qDWS*	3	RM293–RM468	4.49	0.06	12.37	Nguyen et al., 2016
Filled grains per panicle	*qFGPP-2a*	2	RM341-RM6056	9.2	−9.07	2.6	Tong et al., 2010
	*qFGPP-3*	3	RM282-MRG0164	9.8	−17.662	7.3	Tong et al., 2010
	*qFGPP-10b*	10	RM228-RM590	6.6	−5.99	1	Tong et al., 2010
	*qFGPP-12*	12	RM19-RM247	3.9	−9.39	1.9	Tong et al., 2010
	*qFGPP-2b*	2	RM6056-MRG2762	8.1	−8.46	2.4	Tong et al., 2010
	*qFGPP-2d*	2	RM263-RM221	4.2	−13.51	1.3	Tong et al., 2010
	*qFGPP-3*	3	RM282-MRG0164	10.8	−23.36	10.8	Tong et al., 2010
	*qFGPP-4*	4	MRG2558-RM273	6.6	−7.99	2.2	Tong et al., 2010
	*qFGPP-6*	6	RM204-RM225	4	5.86	1.3	Tong et al., 2010
	*qFGPP-10a*	10	RM330A-RM216	3.6	−6.91	1.1	Tong et al., 2010
	*qFGPP-2c*	2	MRG2762-RM3515	6.6	−8.14	2.8	Tong et al., 2010
	*qFGPP-3*	3	RM282-MRG0164	8.8	−20.88	13.3	Tong et al., 2010
	*qFGPP-5*	5	RM437-RM169	3.7	5.85	1	Tong et al., 2010
	*qFGPP-10b*	10	RM228-RM590	4.8	−7.18	1.2	Tong et al., 2010
Grain number per panicle	*qGNPP-2b*	2	RM6056-MRG2762	3.8	−9.39	1.1	Tong et al., 2010
	*qGNPP-3a*	3	RM282-MRG0164	4.5	−18.646	5.5	Tong et al., 2010
	*qGNPP-3b*	3	RM532-RM520	7.6	−11.06	2	Tong et al., 2010
	*qGNPP-1*	1	RM243-RM575	5.8	−8.05	1.3	Tong et al., 2010
	*qGNPP-2b*	2	RM6056-MRG2762	8.5	−10.27	2.1	Tong et al., 2010
	*qGNPP-3a*	3	RM282-MRG0164	11.9	−23.36	10.8	Tong et al., 2010
	*qGNPP-3d*	3	MRG5949-RM293	7.6	−13.1	2.3	Tong et al., 2010
	*qGNPP-7a*	7	RM481-MRG4711	6.7	−8.72	1.5	Tong et al., 2010
	*qGNPP-2a*	2	RM236-RM233B	4.7	2.83	2.1	Tong et al., 2010
	*qGNPP-3a*	3	RM282-MRG0164	8.3	−13.76	8.7	Tong et al., 2010
	*qGNPP-4*	4	RM252-MRG5454	4.5	−7.74	1.5	Tong et al., 2010
	*qGNPP-7b*	7	MRG4499-RM2	9.9	−10.49	2.7	Tong et al., 2010
	*qGNPP-12*	12	RM117-RM101	12.3	−3.24	10.2	Tong et al., 2010
Grain yield	*qRGY3*	3	RM232-C63	3.65	−0.02	10.8	Wei et al., 2012
	qRGY3-1*	3	C63-C316	2.07	−0.01	4	Wei et al., 2012
	*qRGY7*	7	RG678-R1440	2.7	−0.02	8.1	Wei et al., 2012
	*qRGY11*	11	CDO127-R3203	2.65	0.02	7.8	Wei et al., 2012
	*qGYl2-1*	2	G1314a–RZ386	3.11	-0.21	10.77	Wei et al., 2011
	*qGYl2-2*	2	R1843–RM29	2.98	-0.18	7.6	Wei et al., 2011
	*qGYl2-3*	2	RM53–R1738	3.49	-0.20	10.25	Wei et al., 2011
	*qGYl7-1*	7	RZ471–RG678	4	0.22	12.16	Wei et al., 2011
	*qGYl7-2*	7	R1440–C1023	4.27	0.22	12.19	Wei et al., 2011
	*qGYn2-1*	2	RM53–R1738	5.13	-0.34	18.53	Wei et al., 2011
	*qGYn7-1*	7	RZ471–RG678	3.84	0.27	11.34	Wei et al., 2011
	*qGYn7-2*	7	RG678–R1440	4.51	0.29	12.88	Wei et al., 2011
	*qGYn7-3*	7	C1023–RG128	3.18	0.4	24.43	Wei et al., 2011
	*qGYl7-3*	7	RZ471–RG678	4.32	0.29	13.1	Wei et al., 2011
	*qGYl11*	11	R3203–RM20a	2.89	0.24	9.38	Wei et al., 2011
	*qGYn1*	1	C86–C2340	3.14	0.23	9.03	Wei et al., 2011
	*qGYn2-2*	2	RZ599–RM53	3.2	-0.26	12.01	Wei et al., 2011
	*qGYn7-4*	7	RZ471–RG678	3.8	0.25	10.87	Wei et al., 2011
	*qGY6.1*	6	6517443–6942384	8.95	–	6.28	Zhou et al., 2017
	*qGY8.1*	8	2492172–2797908	9.78	–	7.31	Zhou et al., 2017
	*qSPY-3*	3	RZ678-RZ574	5.05	0.73	24.7	Senthilvel et al., 2008
	*qGYPP-7b*	7	CH742-CH743	11.4	−0.95	2.7	Tong et al., 2010
	*qGYPP-3a*	3	MRG4474-RM545	5.3	−0.62	1	Tong et al., 2010
	*qGYPP-4a*	4	MRG5943-RM471	6.5	−1.09	3.2	Tong et al., 2010
	*qGYPP-3b*	3	RM545-MRG4896	7	−0.72	1.3	Tong et al., 2010
	*qGYPP-4b*	4	RM273-RM252	15.9	−0.58	10.9	Tong et al., 2010
	*qGYPP-7a*	7	RM180-CH742	9.7	−0.65	1.1	Tong et al., 2010
Grain yield response	*qGR3*	3	RM232-C63	4.3	−0.13	16.2	Wei et al., 2012
	*qGR9*	9	C472-RM201	3	0.12	12.5	Wei et al., 2012
	*qGR1-1*	1	RM212-R2201	3.63	0.11	12.9	Wei et al., 2012
	*qGR1-2*	1	G393-RG101	3.09	−0.09	9.9	Wei et al., 2012
	*qGR2*	2	RZ599-RM53	2.7	−0.09	9.4	Wei et al., 2012
Number of leaves	*qNL*	3	RM416–RM293	3.38	0.172	9.73	Nguyen et al., 2016
	*qNL*	8	RM042–RM284	5.41	−0.246	17.56	Nguyen et al., 2016
	*qNL*	12	RM453–RM247	3.5	−0.166	8.89	Nguyen et al., 2016
Panicle number per plant	*qPNPP-2*	2	MRG2762-RM3515	6.3	0.49	3.4	Tong et al., 2010
	*qPNPP-6*	6	RM136-RM564	2.9	−0.36	1.9	Tong et al., 2010
	*qPNPP-1a*	1	RM579-RM582	4.6	0.4	2.3	Tong et al., 2010
	*qPNPP-1b*	1	MRG6408-RM212	2.8	−0.34	1.7	Tong et al., 2010
	*qPNPP-2*	2	MRG2762-RM3515	6.9	0.49	3.4	Tong et al., 2010
	*qPNPP-3a*	3	MRG4813-MRG5949	3.9	0.36	1.9	Tong et al., 2010
	*qPNPP-3b*	3	MRG5949-RM293	4.9	0.39	2.6	Tong et al., 2010
	*qPNPP-4a*	4	MRG5454-RM563	2.9	0.26	1.2	Tong et al., 2010
	*qPNPP-4b*	4	RM348-RM131	2.8	0.31	1.5	Tong et al., 2010
Partial factor productivity	*qPFP1.2*	1	SNP_1_23091103	5.89	3.45	13.17	Jewel et al., 2019
	*qPFP2.1*	2	SNP_2_4342883	9.44	−3.99	20.25	Jewel et al., 2019
	*qPFP3.2*	3	SNP_3_3542519	7.32	4.16	16.09	Jewel et al., 2019
	*qPFP4.1*	4	SNP_4_21833014	7.6	3.68	16.66	Jewel et al., 2019
	*qPFP5.2*	5	SNP_5_15469279	9.78	−4.05	20.91	Jewel et al., 2019
	*qPFP6.2*	6	SNP_6_12183428	4.46	2.92	10.14	Jewel et al., 2019
	*qPFP7.2*	7	SNP_7_28303039	7.21	4.04	15.89	Jewel et al., 2019
	*qPFP8.1*	8	SNP_8_322877	7.09	−3.5	15.64	Jewel et al., 2019
	*qPFP9.1*	9	SNP_9_12154616	7.87	4.23	17.19	Jewel et al., 2019
	*qPFP10.1*	10	SNP_10_146531	9.13	−3.92	19.68	Jewel et al., 2019
	*qPFP11.2*	11	SNP_11_2514115	3.66	2.57	8.41	Jewel et al., 2019
Plant height	*qPH*	1	RM265–RM315	6.14	5.046	18.63	Nguyen et al., 2016
	*qPH*	3	RM416–RM293	3.39	3.382	8.91	Nguyen et al., 2016
Relative plant dry weight	*qRPW1*	1	RM5385–RM7192	2.87	0.1	14.45	Feng et al., 2010
	*qRPW8*	8	RM2366–RM5767	2.86	-0.08	10.1	Feng et al., 2010
Relative plant height	*qRPH2*	2	RM240–RM250	2.72	-0.02	9.13	Feng et al., 2010
Relative root length	*qRRL1*	1	RM5385–RM7192	2.72	0.05	10.96	Feng et al., 2010
Relative shoot dry weight	*qRSW1*	1	RM5385–RM7192	2.75	0.08	9.07	Feng et al., 2010
	*qRSW3*	3	RM5626–RM7097	2.95	0.09	12.38	Feng et al., 2010
	*qRSW8*	8	RM2366–RM5767	3.66	-0.08	11.22	Feng et al., 2010
Total dry weight	*qDW*	3	RM293–RM468	4.54	0.169	12.89	Nguyen et al., 2016
Total fresh weight	*qFW*	3	RM293–RM468	5.34	1.51	14.99	Nguyen et al., 2016

**TABLE 2:** Detailed description of QTL associated with nitrogen use efficiency in rice.

Traits	QTL name	Chr	Marker/interval(bp)	LOD/*F* value	ADD	*R*^2^	References
Absorption nitrogen use efficiency	*qaNUE*	3	RM055–RM3199	4.15	0.003	16.07	Nguyen et al., 2016
	*qaNUE*	8	RM433–RM230	5.74	−0.004	25.08	Nguyen et al., 2016
Agricultural nitrogen-absorption efficiency	*qANAE4*	4	RM5757	3.7	2.53	6.7	Dai et al., 2015
	*qANAe5*	5	RM5968	2.3	1.99	4.2	Dai et al., 2015
	*qANAE8*	8	RM5485	2.4	−2.1	4.8	Dai et al., 2015
	*qANAE9*	9	RM6491	2.3	2.06	4.6	Dai et al., 2015
Agricultural nitrogen use efficiency	*qANUE4*	4	RM5757	2.3	1.19	4.4	Dai et al., 2015
Agronomic efficiency	*qAE12.1*	12	SNP_12_14936674	2.55	2.28	10.27	Jewel et al., 2019
Agronomical nitrogen use efficiency	*qagNUE*	3	RM055–RM3199	3.83	0.104	17.47	Nguyen et al., 2016
Biomass nitrogen	*qRBN2*	2	RZ599-RM53	3.2	−0.02	8.9	Wei et al., 2012
	*qRBN9*	9	RG667-RG570	3.57	−0.02	9.4	Wei et al., 2012
	qRBN2-1*	2	RZ599-RM53	2.02	−0.01	6.3	Wei et al., 2012
	*qRBN4-1*	4	R78-G235	2.7	0.02	8.4	Wei et al., 2012
	*qRBN4-2*	4	G235-G102	4.59	0.03	14.6	Wei et al., 2012
Grain nitrogen	qRGN1-1*	1	RG101-C922	1.71	−0.01	4.8	Wei et al., 2012
	*qRGN9*	9	RG667-RG570	3.11	−0.02	8.6	Wei et al., 2012
	*qRGN1*	1	G393-RG101	3.41	−0.03	13.2	Wei et al., 2012
Nitrogen absorption ability	*qNAA12*	12	RM5364	2.4	0.02	4.3	Dai et al., 2015
	*qNAA4*	4	RM5757	3.2	0.02	5.7	Dai et al., 2015
	*qNAA5*	5	RM5968	2.9	0.02	5	Dai et al., 2015
	*qNAA10*	10	RM6142	4.1	−0.02	8.6	Dai et al., 2015
Nitrogen concentration in roots	*qNR*	1	RM579–RM312	3.1	−0.113	19.83	Nguyen et al., 2016
	*qNR*	1	RM104–RM129	5.14	−0.129	25.64	Nguyen et al., 2016
	*qNR*	1	RM472–RM431	4.03	0.116	18.55	Nguyen et al., 2016
	*qNR*	8	RM337	4.49	−0.108	21.39	Nguyen et al., 2016
	*qNR*	11	RM120–RM479	4.24	0.119	22.34	Nguyen et al., 2016
Nitrogen concentration in shoots	*qNS*	11	RM004b–RM332	3.26	−0.174	22.98	Nguyen et al., 2016
Nitrogen harvest index	*qNHI12*	12	RM7003	2.7	0.02	5.3	Dai et al., 2015
	*qNHI2*	2	RM5812	2.4	0.01	4.7	Dai et al., 2015
Nitrogen response	*qNR6*	6	RZ398-C764	5.48	−4.68	16.6	Wei et al., 2012
	*qNR10*	10	R2625-RG561	2.8	3.04	7.5	Wei et al., 2012
	*qNR4*	4	G235-G102	3.65	3.26	11.01	Wei et al., 2012
Nitrogen uptake	*qNUP2.1*	2	36017977–36777825	8.86	–	3.83	Zhou et al., 2017
	*qNUP3.1*	3	25056241–25069454	9.64	–	4.75	Zhou et al., 2017
	*qNUP6.1*	6	7814673–9668398	19.68	–	11.86	Zhou et al., 2017
	*qNUP8.1*	8	2797908–3336084	20.16	–	13.99	Zhou et al., 2017
	*qNUP10.1*	10	22335288–22517954	18.57	–	9.8	Zhou et al., 2017
	*qNUP11.1*	11	19120157–19494142	7.73	–	3.16	Zhou et al., 2017
	*qNUP11.2*	11	25559185–26317711	9.34	–	4.3	Zhou et al., 2017
	*qNUE2.1*	2	31531953–32386052	6.96	–	3.98	Zhou et al., 2017
	*qNUE4.1*	4	23285463–23315504	7.34	–	4.4	Zhou et al., 2017
	*qNUE6.1*	6	6517443–6942384	17.46	–	12.34	Zhou et al., 2017
	*qNUE6.2*	6	9668398–9927733	7.59	–	4.79	Zhou et al., 2017
	*qNUE10.1*	10	17355105–17376675	6.9	–	3.76	Zhou et al., 2017
	*qNUE10.2*	10	20364788–20798359	8.84	–	5.87	Zhou et al., 2017
Nitrogen use efficiency	*qNUEl2-1*	2	RM53–R1738	5.36	-3.20	21.62	Wei et al., 2011
	*qNUEl6*	6	R2749–R1952a	3.6	-2.93	13.25	Wei et al., 2011
	*qNUEn1*	1	C86–C2340	3.8	1.71	11.17	Wei et al., 2011
	*qNUEn2-1*	2	RM53–R1738	3.15	-1.90	14.85	Wei et al., 2011
	*qNUEl2-2*	2	RZ599–RM53	3.06	-2.74	11.46	Wei et al., 2011
	*qNUEl7-1*	7	RZ471–RG678	4.44	2.76	11.35	Wei et al., 2011
	*qNUEl7-2*	7	R1440–C1023	2.94	2.45	9.09	Wei et al., 2011
	*qNUEl11*	11	R3203–RM20a	2.8	2.24	6.8	Wei et al., 2011
	*qNUEn2-2*	2	RM53–R1738	3.68	-1.69	14.03	Wei et al., 2011
	*qNUEn11*	11	C1237–RG118	3.25	1.64	11.14	Wei et al., 2011
	*qNUE-3*	3	RZ574-RZ284	5.46	4.11	26.4	Senthilvel et al., 2008
Percent N content	*qNCP-3-1*	3	RG191-RZ678	6.12	−0.03	29.1	Senthilvel et al., 2008
	*qNCP-3-2*	3	Pgi1-CDO87	4.67	−0.03	23.6	Senthilvel et al., 2008
Physiological nitrogen use efficiency	*qpNUE*	11	RM287–RM209	3.08	−0.899	18.17	Nguyen et al., 2016
	*qPE3*	3	RM232-C63	4.14	−2.13	12.8	Wei et al., 2012
	qPE3-1*	3	C63-C316	2.46	−1.45	5.6	Wei et al., 2012
	*qPE7*	7	RG678-R1440	3.01	−1.86	8.8	Wei et al., 2012
	*qPE11*	11	C1237-RG118	2.98	2.77	9.3	Wei et al., 2012
	*qPNUE3*	3	RM5761	2.5	2.22	6.5	Dai et al., 2015
	*qPNUE4*	4	RM1205	2.5	1.81	4.5	Dai et al., 2015

**TABLE 3:** Detailed description of QTL associated with nitrogen use efficiency-related traits in wheat.

Traits	QTL name	Chr	Marker/interval	LOD	ADD	*R*^2^	References
1000 kernel weight	*QTkw_3B*	3B	TC249615-Xgwm376.2	–	–	–	Xu et al., 2013
	*QTkw_4B*	4B.1	Xlhq145-Xdupw619	–	–	–	Xu et al., 2013
	*QTkw_4D*	4D	Xcfd193-Xcfd71	–	–	–	Xu et al., 2013
	*QTkw_2D*	2D	gwm102	4.7	–	10.2	Laperche et al., 2007a
	*QTkw_5A*	5A	cfa2149	3.9	–	17.6	Laperche et al., 2007a
	*QTkw_5B*	5B	wmc339	3.3	–	3.29	Laperche et al., 2007a
	*QTkw_7B*	7B	gpw3215b	3.6	–	5.7	Laperche et al., 2007a
	*QTkw_2DL*	2DL	gpw4085	5.91	−0.13	14.6	Zheng et al., 2010
	*QTkw_6D*	6D	cfd80	24.5	−2.99	13.5	Habash et al., 2007
	*QTkw_2B*	2B	barc101a	21.7	−1.08	4.1	Habash et al., 2007
	*QTkw_4A*	4A	m92p78.8	39.2	1.64	7.8	Habash et al., 2007
	*QTkw_4B*	4B	Rht-B1	34.2	1.40	6.5	Habash et al., 2007
	*QTkw_5A*	5A	barc141	26.1	−1.17	4.9	Habash et al., 2007
	*QTkw_6A*	6A	rsq805.1	23.7	−1.18	4.2	Habash et al., 2007
	*QTkw_6B*	6B	psp3118	74.3	−3.48	20.5	Habash et al., 2007
	*QTkw_6D*	6D	psr899.2	33.1	1.47	6.3	Habash et al., 2007
	*QTkw_7B*	7B	m62p64.9	23	1.14	4.1	Habash et al., 2007
	*QTkw_7D*	7D	barc26	29.3	−1.27	5.3	Habash et al., 2007
	*TKW4_9*	1B	WMC500B-CFD48	3.46	–	0.09	Cormier et al., 2016
	*TKW5*	1B	WPT0506-WPT0419	3.5	–	0.09	Cormier et al., 2016
	*TKW2*	3B	CFB3260-CFB3260	3.09	–	0.1	Cormier et al., 2016
	*TKW10*	4A	GPW2244-WPT2006	3.09	–	0.07	Cormier et al., 2016
	*TKW7*	5B	CDO584-WPT0517	3.1	–	0.07	Cormier et al., 2016
	*TKW3*	6A	GWM427-TPT4178	4.12	–	0.12	Cormier et al., 2016
	*TKW6*	6A	WPT0938-TPT4178	4.12	–	0.1	Cormier et al., 2016
	*TKW1*	7B	WPT4230-BARC315	4.4	–	0.09	Cormier et al., 2016
	*QTkw.sdau-3A-1*	3A	Xwmc264	3.87	1.47	10.57	Deng et al., 2017
	*QTkw.sdau-6A*	6A	Xbarc1055	5.11	1.50	10.88	Deng et al., 2017
	*QTkw.sdau-3A-1*	3A	Xwmc264	3.61	1.56	8.75	Deng et al., 2017
	*QTkw.sdau-5B*	5B	Xgwm213	3.3	-1.39	6.43	Deng et al., 2017
	*QTkw.sdau-6A*	6A	Xbarc1055	3.92	1.48	7.8	Deng et al., 2017
	*QTkw.sdau-5B*	5B	Xgwm213	3.16	-1.46	6.03	Deng et al., 2017
	*QTkw.sdau-6A*	6A	Xbarc1055	3.01	1.39	5.87	Deng et al., 2017
	*QTkw.sdau-3A-1*	3A	Xwmc264	5.82	1.71	8.99	Deng et al., 2017
	*QTkw.sdau-4D*	4D	Xbarc334	3.23	1.24	4.71	Deng et al., 2017
	*QTkw.sdau-5B*	5B	Xgwm213	3.01	-1.21	4.13	Deng et al., 2017
	*QTkw.sdau-6A*	6A	Xbarc1055	9.35	2.24	15.18	Deng et al., 2017
	*QTkw.sdau-7D-1*	7D	Xgwm676	4.1	1.56	7.00	Deng et al., 2017
	*QTkw.sdau-7D-2*	7D	Xgdm67	5.87	-1.73	9.18	Deng et al., 2017
	*QTkw.sdau-2B-1*	2B	Xwmc179	8.77	2.08	15.93	Deng et al., 2017
	*QTkw.sdau-2B-2*	2B	Xbarc1042	3.25	-1.24	5.66	Deng et al., 2017
	*QTkw.sdau-2D*	2D	Xwmc170.2	4.92	1.54	8.61	Deng et al., 2017
	*QTkw.sdau-6A*	6A	Xbarc1055	6.04	1.77	11.41	Deng et al., 2017
	*QTkw.sdau-1A*	1A	Xcfd59	3.16	1.21	4.44	Deng et al., 2017
	*QTkw.sdau-3A-2*	3A	Xbarc51	3.65	1.28	5.23	Deng et al., 2017
	*QTkw.sdau-4D*	4D	Xbarc334	3.77	1.29	5.27	Deng et al., 2017
	*QTkw.sdau-5B.2*	5B	Xbarc140	5.47	-1.57	7.80	Deng et al., 2017
	*QTkw.sdau-6A*	6A	Xbarc1055	4.19	1.50	7.04	Deng et al., 2017
	*QTkw.sdau-6D*	6D	Xcfd13	5.68	-1.68	8.85	Deng et al., 2017
	*QTkw.sdau-6A*	6A	Xbarc1055	5.86	2.10	14.64	Deng et al., 2017
	*QTkw.sdau-2D*	2D	Xwmc170.2	3.1035	1.36	6.82	Deng et al., 2017
	*QTkw.sdau-6A*	6A	Xbarc1055	4.98	1.81	12.09	Deng et al., 2017
	*QTkw.sdau-1B*	1B	Xwmc766	7.09	1.91	21.03	Deng et al., 2017
	*QTkw.sdau-4B*	4B	Xwmc413	9.93	1.63	19.18	Deng et al., 2017
	*QTkw.sdau-5B.2*	5B	Xbarc140	3.21	-0.88	5.56	Deng et al., 2017
	*QTkw.sdau-6D*	6D	Xcfd13	4.85	-1.19	10.18	Deng et al., 2017
	*QTkw.sdau-1B*	1B	Xwmc766	3.1	1.05	10.01	Deng et al., 2017
	*QTkw.sdau-2B-3*	2B	Xcwem55	3.85	-1.02	8.51	Deng et al., 2017
	*QTkw.sdau-6A*	6A	Xbarc1055	3.63	1.00	8.13	Deng et al., 2017
Dry matter grain yield	*DMGY9*	1B	CDO346-CDO346	3.14	–	0.07	Cormier et al., 2016
	*DMGY11*	3A	WPT1816-GWM666B	4.09	–	0.01	Cormier et al., 2016
	*DMGY6*	3A	WPT6234-WPT6234	4.15	–	0.02	Cormier et al., 2016
	*DMGY12*	5A	GWM241-GWM241	3.82	–	0.02	Cormier et al., 2016
	*DMGY2*	6A	GPW3251-GPW3251	3.38	–	0.18	Cormier et al., 2016
	*DMGY3*	6A	PTAG53-WPT0562	3.03	–	0.17	Cormier et al., 2016
	*DMGY1*	7B	WPT7113-BARC182	3.1	–	0.15	Cormier et al., 2016
	*DMGY10*	7D	GPW334-GPW334	3.82	–	0.07	Cormier et al., 2016
Ear number/plant	*ENP*	1B	csu109	34.8	1.01	6.9	Habash et al., 2007
	*ENP*	1B	psr967.2	28.1	−0.70	5.3	Habash et al., 2007
	*ENP*	2B	m72p78.8	39.9	−0.86	10	Habash et al., 2007
	*ENP*	3D	wmc533	24.7	0.66	5.6	Habash et al., 2007
	*ENP*	4B	gwm513	52.8	−1.97	19.8	Habash et al., 2007
	*ENP*	4B	m65p64.8	52.2	0.98	11.3	Habash et al., 2007
	*ENP*	5B	dupw115	27.6	0.61	5.2	Habash et al., 2007
	*ENP*	6B	wmc397	54.2	1.01	11.6	Habash et al., 2007
Grain filling duration	*GPD*	1D	wmc429	21.6	1.29	9.4	Habash et al., 2007
	*GPD*	4B	psp3030.2	45.1	−1.52	14.2	Habash et al., 2007
	*GPD*	4D	gwm165.2	21.1	0.97	5.8	Habash et al., 2007
	*GPD*	4D	psr375.1	20.3	−0.87	5.7	Habash et al., 2007
	*GPD*	5D	cfd3	53.9	1.59	17.1	Habash et al., 2007
Grain nitrogen	*GN*	1A	wmc278	26.3	−0.04	5.1	Habash et al., 2007
	*GN*	1A	m92p78.4	31.3	−0.05	6.7	Habash et al., 2007
	*GN*	2A	m92p78.10	32.4	0.05	7.9	Habash et al., 2007
	*GN*	2A	wmc453a	29.8	−0.04	5.9	Habash et al., 2007
	*GN*	2B	barc101a	35	−0.05	7.3	Habash et al., 2007
	*GN*	4A	wmc313	26.4	0.04	5.2	Habash et al., 2007
	*GN*	5B	wmc149a	43.5	−0.05	9.4	Habash et al., 2007
	*GN*	5D	m77p64.8	22.6	0.06	4.8	Habash et al., 2007
	*GN*	6B	m87p78.5a	77.4	−0.07	19.6	Habash et al., 2007
	*GN*	6D	psr899.2	22.6	0.04	4.8	Habash et al., 2007
	*GN*	7D	awm448	29	0.05	7.2	Habash et al., 2007
Grain nitrogen content	*QGnc*	6A	Xcfd80.2–Xbarc1055	–	0.81	9.4	Xu et al., 2013
	*GNE*	3B	wmc326	44.2	−2.21	8.5	Habash et al., 2007
	*GNE*	4A	m68p78.x	22.8	1.41	3.8	Habash et al., 2007
	*GNE*	4B	Rht-B1	41.6	2.00	8.4	Habash et al., 2007
	*GNE*	5A	vrn-A1	22.5	−1.49	4.2	Habash et al., 2007
	*GNE*	5D	m63p78.1b	22	−1.42	3.9	Habash et al., 2007
	*GNE*	6A	m62p64.12	36.4	−2.11	7.6	Habash et al., 2007
	*GNE*	6B	m87p78.5a	83	−3.27	19	Habash et al., 2007
	*GNE*	6D	cfd80	41.9	−2.66	13.2	Habash et al., 2007
	*GNE*	7B	wmc76	30.4	−1.74	5.7	Habash et al., 2007
	*GNP*	4D	gwm165.2	25.3	−0.02	19.5	Habash et al., 2007
	*GNP*	6D	m69p78.10	24.1	−0.01	10.8	Habash et al., 2007
Grain nitrogen percent	*GN*	2A	m92p78.10	34.4	0.11	9.3	Habash et al., 2007
	*GN*	4A	psp3028	34.8	0.09	10.4	Habash et al., 2007
	*GN*	5B	m51p65.4	41	−0.17	10.6	Habash et al., 2007
	*GN*	5D	psr806.3	38.2	−0.09	9.7	Habash et al., 2007
	*GN*	5D	p77p64.8	68.6	0.15	21.3	Habash et al., 2007
	*GN*	7A	psp3050	24.5	0.07	5.8	Habash et al., 2007
Grain nitrogen yield	*GNY4*	1B	WPT1972-WMC419	3.75	–	0.06	Cormier et al., 2016
	*GNY6*	1B	KSUI27B-WPT3177	3.19	–	0.08	Cormier et al., 2016
	*GNY8*	1B	WPT1973-WPT1973	3.28	–	0.07	Cormier et al., 2016
	*GNY7*	1D	WPT8854-GPW300	4.42	–	0.1	Cormier et al., 2016
	*GNY2*	2A	WMC326-GPW5257	5.29	–	0.14	Cormier et al., 2016
	*GNY3*	3A	WPT9268-WMC169	3.74	–	0.07	Cormier et al., 2016
	*GNY11*	4B	GWM573-WPT8756	3.11	–	0.03	Cormier et al., 2016
	*GNY9*	5A	ABG366-ABG366	3.48	–	0.08	Cormier et al., 2016
	*GNY5*	6A	CFE80-GPW7455	3.93	–	0.07	Cormier et al., 2016
	*GNY1*	7A	WMC488-WPT2083	3.49	–	0.05	Cormier et al., 2016
	*GNY10*	7B	WPT5463-STM5TCACA	3.02	–	0.07	Cormier et al., 2016
Grain number per ear	*GNE*	3B	m21p76.3	23.5	−4.69	7.6	Habash et al., 2007
Grain protein concentration	*GPC4*	3A	CDO482-CDO482	3.44	–	−0.01	Cormier et al., 2016
	*GPC7*	3B	WMC540-WMC540	3.09	–	0.13	Cormier et al., 2016
	*GPC9*	4A	WPT5172-WPT2780	3.3	–	0.04	Cormier et al., 2016
	*GPC6*	5A	WG564-PSB85	3.92	–	0.23	Cormier et al., 2016
	*GPC2*	5B	WPT6726-DUPW395	3.27	–	0.27	Cormier et al., 2016
	*GPC8*	6D	WPT1519-WPT672044	4.47	–	0.2	Cormier et al., 2016
	*GPC5*	7B	BE499017-WMC546C	3.03	–	0.12	Cormier et al., 2016
Grain protein content	*QGPA*	3A	gwm666a	3.4	–	5.3	Laperche et al., 2007a
	*QGPC*	1A	Gpw2277	3.6	–	7.5	Laperche et al., 2007a
	*QGPC*	2A	cfa2043b	3.6	–	10	Laperche et al., 2007a
	*QGPC*	4B	gwm367b	3.2	–	9.5	Laperche et al., 2007a
Grain protein content, grain number	*QGPC.QGPA*	3D	cfd223	4.2	–	9.9	Laperche et al., 2007a
Grain protein content, grain yield	*QGPA,QGY*	3D	cfd9	4	–	7.9	Laperche et al., 2007a
Grain weight	*GNE*	1A	psr967.1	23.8	−0.08	4.7	Habash et al., 2007
	*GNE*	1D	cfd65a	44.3	0.18	18.3	Habash et al., 2007
	*GNE*	3A	wmc532	20	−0.09	5.8	Habash et al., 2007
	*GNE*	4B	psp3163	87.3	0.22	28	Habash et al., 2007
	*GNE*	5B	m77p64.3	25.9	−0.10	5.2	Habash et al., 2007
	*GNE*	5D	GS2-related	54.4	0.18	15.8	Habash et al., 2007
	*GNE*	6B	wmc397	72.6	−0.18	20.5	Habash et al., 2007
	*GNE*	6D	m63p78.8	33.8	−0.10	7	Habash et al., 2007
	*GNE*	7A	wmc422	50.2	−0.14	11.5	Habash et al., 2007
	*GNE*	7B	psr927.1	20.1	−0.08	4.4	Habash et al., 2007
	*GNP*	2B	gwm210.1	20.3	−0.87	5.4	Habash et al., 2007
	*GNP*	2D	gwm30.1	26.1	−1.06	7.6	Habash et al., 2007
	*GNP*	4D	psr375.1	40.9	−1.81	22.4	Habash et al., 2007
	*GNP*	6D	m69p78.10	39.6	−1.37	15.2	Habash et al., 2007
	*GNP*	6Da	p69p78.10	39.8	−1.52	18.3	Habash et al., 2007
	*GNP*	7A	psp3050	31.9	−1.22	9.2	Habash et al., 2007
	*GNP*	7B	psr927.1	44.1	−1.44	14.2	Habash et al., 2007
	*GNP*	7B	m43p78.14	36.7	1.82	24.4	Habash et al., 2007
Grain yield	*QGY*	5A	gpw3124	3.9	–	8.8	Laperche et al., 2007a
	*QGY*	5A	gwm639b	2.9	–	5.7	Laperche et al., 2007a
	*QGY*	2A2	cfa2043b	3.06	–	6.5	Laperche et al., 2007a
	*QGY*	2D1	gpw4085	3.31	–	6.6	Laperche et al., 2007a
	*QGY*	3D	cfd9	4.22	–	7.9	Laperche et al., 2007a
	*QGY*	4B	wmc238	3.94	–	7.5	Laperche et al., 2007a
	*QGY*	4B	rht	3.57	–	9.6	Laperche et al., 2007a
	*QGY*	5A1	gwm639b	2.93	–	5.7	Laperche et al., 2007a
	*QGY*	2D1	gwm484	7.26	–	17.6	Laperche et al., 2007a
	*QGY*	3D	cfd9	3.89	–	7.1	Laperche et al., 2007a
	*QGY*	4B	Rht-b1	3.36	–	7.9	Laperche et al., 2007a
	*QGY*	5A1	gwm639b	3.04	–	4.8	Laperche et al., 2007a
Grain yield, grain protein content	*QNH1, QNSA, QGY, QNTOT, QGPA, QGPC*	2D	gwm484	4.4,3.9	–	13.6	Laperche et al., 2007a
Harvest index	*QHi*	4B	Xgwm192.1–Xbarc20	–	0.01	12.2	Xu et al., 2013
Kernel number	*QKN*	2D1	gpw4085	7.07	–	13	Laperche et al., 2007a
	*QKN*	2D1	gpw4085	3.55	–	6	Laperche et al., 2007a
	*QKN*	3A	gwm66a	3.39	–	5.3	Laperche et al., 2007a
	*QKN*	3D	cfd9	3.83	–	6.8	Laperche et al., 2007a
	*QKN*	3D	cfd223	4.77	–	9.9	Laperche et al., 2007a
	*QKN*	3D	cfd223	4.23	–	7.1	Laperche et al., 2007a
	*QKN*	4B	rht	4.89	–	13.1	Laperche et al., 2007a
	*QKN*	4B	rht	5.68	–	8.9	Laperche et al., 2007a
	*QKN*	4B	rht	16.51	–	32.6	Laperche et al., 2007a
	*QKN*	4B	rht	17.42	–	33	Laperche et al., 2007a
	*QKN*	4B	rht	4.11	–	9.9	Laperche et al., 2007a
	*QKN*	4B	rht	17.17	–	32.6	Laperche et al., 2007a
	*QKN*	4B	gpw1108	10.66	–	19.8	Laperche et al., 2007a
	*QKN*	4B	rht	10.42	–	23.7	Laperche et al., 2007a
	*QKN*	4B	gwm495	5.05	–	10.6	Laperche et al., 2007a
	*QKN*	2DL	fdgogatD	12.3	–	27.2	Laperche et al., 2007a
	*QKN*	2DL	gpw4085	13.63	–	24.7	Laperche et al., 2007a
	*QKN*	2DL	gpw4085	6	–	11.1	Laperche et al., 2007a
	*QKN*	3D	cfd9	4.4	–	7.1	Laperche et al., 2007a
	*QKN*	3D	gwm314	3.02	–	5.4	Laperche et al., 2007a
	*QKN*	4B	gwm637b	3.75	–	8.86	Laperche et al., 2007a
	*QKN*	4B	rht-b1	9.2	–	20.3	Laperche et al., 2007a
	*QKN*	4B	wmc238	3.17	–	8.3	Laperche et al., 2007a
	*QKN*	4B	rht-b1	4.41	–	28.1	Laperche et al., 2007a
	*QKN*	4B	rht-b1	5.41	–	10.7	Laperche et al., 2007a
Kernel number, nitrogen nutrition index	*KN, NNI*	1B	gwm268	2.7	0.45	5.8	Zheng et al., 2010
	*KN, NNI*	2BL	gwm429	3.4	0.60	10.8	Zheng et al., 2010
	*KN, NNI*	2DL	gpw4085	2.8	0.54	7.3	Zheng et al., 2010
	*KN, NNI*	4B	wmc238	14.2	−1.06	26.8	Zheng et al., 2010
Kernel weight per spike	*QKws*	6A	Xcfd80.2–Xbarc1055	–	−0.06	9.3	Xu et al., 2013
Leaf fresh weight	*qlfw*	1A	m71p78.5	29.9	−0.09	5.5	Habash et al., 2007
	*qlfw*	1B	m43p78.7	23.2	−0.06	5.1	Habash et al., 2007
	*qlfw*	2A	m83p65.2	52.9	−0.12	12	Habash et al., 2007
	*qlfw*	3A	psr345.2	47	0.08	8.2	Habash et al., 2007
	*qlfw*	3A	cfa2234	33.8	−0.06	5.6	Habash et al., 2007
	*qlfw*	4A	psr593.2	21.4	−0.07	4.3	Habash et al., 2007
	*qlfw*	4A	gwm165.3	33.3	0.08	7.2	Habash et al., 2007
	*qlfw*	5A	psr967.3	49.7	−0.08	9.4	Habash et al., 2007
	*qlfw*	5D	gwm212	59.8	0.10	13.1	Habash et al., 2007
	*qlfw*	7A	psp3001.1	20.1	−0.04	3.1	Habash et al., 2007
	*qlfw*	7D	mgl59	24.4	−0.05	4.4	Habash et al., 2007
Max root length	*QdMrl-2B*	2B	-	4.15	1.02	9.91	Fan et al., 2018
	*QdMrl-4B*	4B	-	4.88	1.44	12.24	Fan et al., 2018
	*QdMrl-7D*	7D	-	4.42	3.61	18.56	Fan et al., 2018
	*qMRL.LN-2B*	2B	Xgwm210-Xbarc1138.2	6.5	4.70	21.6	Ren et al., 2017
	*qMRL.LN-5A*	5A	Xgwm443.1-Xcfa21041	2.7	-2.7	6.8	Ren et al., 2017
Number of grains per spike	*QGns.sdau-4A-2*	4A	Xwmc497	3.29	-1.50	6.77	Deng et al., 2017
	*QGns.sdau-2B*	2B	Xwmc179	5.56	-1.94	13.77	Deng et al., 2017
	*QGns.sdau-2B*	2B	Xwmc179	6.22	-1.92	17.15	Deng et al., 2017
	*QGns.sdau-4A-1*	4A	Xwmc718	4.72	-1.34	10.57	Deng et al., 2017
	*QGns.sdau-2B*	2B	Xwmc179	7.24	-1.74	17.24	Deng et al., 2017
	*QGns.sdau-2B*	2B	Xwmc179	4.78	-1.14	9.36	Deng et al., 2017
	*QGns.sdau-2D*	2D	Xbarc349.2	3.28	1.04	7.46	Deng et al., 2017
	*QGns.sdau-2B*	2B	Xwmc179	3.2	-1.79	7.15	Deng et al., 2017
	*QGns.sdau-4A-1*	4A	Xwmc718	4.75	-2.24	11.24	Deng et al., 2017
	*QGns.sdau-1A*	1A	Xcfd59	5.14	2.00	9.83	Deng et al., 2017
	*QGns.sdau-2B*	2B	Xwmc179	3.92	-1.71	7.42	Deng et al., 2017
	*QGns.sdau-3B*	3B	Xgwm566	3.49	1.67	6.75	Deng et al., 2017
	*QGns.sdau-3D*	3D	Xcfd223	3.22	1.73	7.53	Deng et al., 2017
	*QGns.sdau-7A*	7A	Xwmc530	3.74	1.70	7.22	Deng et al., 2017
Number of root tips	*QdRt-7A*	7A		4.36	26.09	7.67	Fan et al., 2018
Peduncle nitrogen	*QPN*	1B	barc152	65.7	0.14	10	Habash et al., 2007
	*QPN*	1B	m92p78.2	24.7	0.07	2.8	Habash et al., 2007
	*QPN*	2B	psr1870	36.2	−0.09	5.4	Habash et al., 2007
	*QPN*	3D	gwm341	46.3	−0.10	6.9	Habash et al., 2007
	*QPN*	5A	vrn-A1	25.4	−0.07	3.1	Habash et al., 2007
	*QPN*	5B	gwm499	21.9	−0.07	2.7	Habash et al., 2007
	*QPN*	5D	cfd3	54.4	0.12	7.2	Habash et al., 2007
	*QPN*	6B	m87p78.5a	112.4	−0.22	19.9	Habash et al., 2007
	*QPN*	7A	m68p78.6	21.9	−0.06	2.5	Habash et al., 2007
	*QPNp*	1A	psr325.1	33.7	−0.07	12	Habash et al., 2007
	*QPNp*	1D	cfd65a	25.4	−0.05	4.4	Habash et al., 2007
	*QPNp*	2A	m87p78.3	24.9	−0.04	4.7	Habash et al., 2007
	*QPNp*	2B	wmc25b	33.7	0.05	6.9	Habash et al., 2007
	*QPNp*	3D	cfd35	37.9	0.06	7.7	Habash et al., 2007
	*QPNp*	4A	m92p78.8	59.4	−0.08	11.6	Habash et al., 2007
	*QPNp*	4B	Rht-B1	73.3	−0.09	16.2	Habash et al., 2007
	*QPNp*	5A	wmc388b	26.3	−0.05	4.2	Habash et al., 2007
	*QPNp*	5D	cfd18	41.5	0.08	7.3	Habash et al., 2007
	*QPNp*	5D	gwm212	32.9	0.07	6.1	Habash et al., 2007
Plant height	*QPH*	3B	psr567.2	21	−5.88	3.6	Habash et al., 2007
	*QPH*	4B	Rht-B1	102.8	15.19	30.5	Habash et al., 2007
	*QPH*	7A	wmc479	20.6	6.01	5.3	Habash et al., 2007
	*QPh*	2D	Xcfd53–Xwmc112	–	−4.25	17.1	Xu et al., 2013
	*QPh*	4B	Xbarc20–Xbarc90	–	−6.04	26.9	Xu et al., 2013
Root dry weight	*QdRd-6D*	6D		4.17	40.77	6.17	Fan et al., 2018
	*QdRd-7A*	7A		3.89	2.34	9.33	Fan et al., 2018
	*QdRdw-7A*	7A		3.72	6.95	5.66	Fan et al., 2018
	*qRDW.LN-4B*	4B	Xbarc90-Xbarc20	4.7	-0.009	10.4	Ren et al., 2017
	*qRDW.LN-4D*	4D	Xgwm165.2-TC237440	2.9	0.01	8.8	Ren et al., 2017
	*qRDW.LN-6A*	6A	Xbarc104-Xdwpw167.3	3	-0.008	8.6	Ren et al., 2017
	*QRdw.1*	1A	wPt-731490-wPt-6358	3.5–8.7	1.41	16.7	Sun et al., 2013
	*QRdw*	2B	wPt-0100-wPt-6627	3.3–5.6	−1.31	12	Sun et al., 2013
Root fresh weight	*QRfw*	1A	wPt-731490-wPt-6358	3.3–9.6	21.40	15.8	Sun et al., 2013
	*QRfw*	1D	wmc432b-wPt-666067	3.3–6.6	−14.79	14.4	Sun et al., 2013
	*QRfw.1*	2B	wPt-0100-wPt-6627	3.7–4.8	−17.01	10.9	Sun et al., 2013
	*QRfw*	2D	wPt-3757-wPt-667054	3.2–5.6	−48.59	12.9	Sun et al., 2013
	*QRfw.1*	4A	srap18-issr23b	4.7–16.1	159.71	38.8	Sun et al., 2013
Root length	*QdRl-3B*	3B	-	4.57	-97.07	14.31	Fan et al., 2018
	*QdRl-6D*	6D	–	4.46	1.39	9.64	Fan et al., 2018
	*QdRl-7D*	7D	–	3.9	-106.14	13.79	Fan et al., 2018
	*QdRl-1D*	1D	–	4.05	100.59	12.13	Fan et al., 2018
Root surface area	*QdRs-6B*	6B	–	3.56	3.97	9.72	Fan et al., 2018
	QdRs-6D*	6D	–	4.58	3.13	12.58	Fan et al., 2018
	*QdRs-7A*	7A	–	3.8	3.06	14.43	Fan et al., 2018
Root/shoot dry weight ratio	*QRsdw.1*	2B	wmc154a-wmc154b	3.5–5.6	−0.12	16.6	Sun et al., 2013
	*QRsdw.2*	2B	wPt-0100-wPt-6627	3.0–7.8	−0.01	6.6	Sun et al., 2013
	*QRsdw.2*	2D	wPt-3757-wPt-667054	4.1–6.5	−0.01	12.3	Sun et al., 2013
Root/shoot fresh weight ratio	*QRsfw.1*	1A	wPt-731490-wPt-6358	4.3–5.0	0.03	9.1	Sun et al., 2013
	*QRsfw*	2B	wPt-0100-wPt-6627	3.0–7.8	−0.03	13.8	Sun et al., 2013
	*QRsfw.2*	2D	wPt-3757-wPt-667054	4.2–4.4	−0.11	20	Sun et al., 2013
	*QRsfw.1*	4A	srap18-issr23b	19.0–30.0	0.47	47.9	Sun et al., 2013
Shoot dry weight	*QSdw.1*	1A	wPt-731490-wPt-6358	3.8–4.3	3.60	12.3	Sun et al., 2013
	*QSdw*	1D	wmc432b-wPt-666067	3.7–5.4	−4.11	15.5	Sun et al., 2013
	*QSdw.1*	2B	wmc154a-wmc154b	3.3–5.9	3.35	10.5	Sun et al., 2013
Shoot fresh weight	*QSfw*	1A	wPt-731490-wPt-6358	–	18.80	13.2	Sun et al., 2013
	*QSfw*	1D	wmc432b-wPt-666067	3.1–6.1	−31.06	15.3	Sun et al., 2013
	*QSfw.1*	2B	wmc154a-wmc154b	3.2–5.9	43.75	13.4	Sun et al., 2013
	*QSfw.2*	2B	wPt-0100-wPt-6627	3.2–5.9	−17.01	10.1	Sun et al., 2013
	*QSfw.1*	4A	srap18-issr23b	4.4–5.6	38.65	6.1	Sun et al., 2013
Spike length	*QSL*	2D	Xcfd53–Xwmc112	–	−0.55	30.4	Xu et al., 2013
	*QSL*	5B.2	Xgwm272–Xswes14	–	0.30	3	Xu et al., 2013
	*QSL*	6D.1	Xcfd80.1–Xgdm14.4	–	−0.27	5.7	Xu et al., 2013
Spike number per plant	*QScn*	2D	Xcfd53–Xwmc112	–	0.20	29.9	Xu et al., 2013
	*QScn*	5B.1	Xgwm133.2–Xwmc73	–	−0.15	5.5	Xu et al., 2013
Straw nitrogen, number of grains per area (m2), grain yield, grain protein yield, thousand Kernel weight	*QNS, QGPA, QGY, QGPY, QTKW*	4B	wmc238	5.8,5.1	–	19.8	Laperche et al., 2007a
Sterile spikelet per spike	*QSss*	2D	Xwmc112-Xbarc168	–	−0.39	11.4	Xu et al., 2013
Total amount of nitrogen, grain protein content	*QNTOT, QGPC*	3B	cfa2170b	3.3	–	8.1	Laperche et al., 2007a
Total amount of nitrogen, thousand Kernel weight, nitrogen amount in the straw, straw nitrogen, number of grains per area	*QNTOT, QGPA, QTKW, QNS, QNSA*	4B	rht-B1	9.4,8.4	–	33	Laperche et al., 2007a
Total dry weight	*QTdw.1*	1A	wPt-731490-wPt-6358	3.6–5.6	4.47	13	Sun et al., 2013
	*QTdw*	1D	wmc432b-wPt-666067	3.6–4.3	−5.14	13.3	Sun et al., 2013
	*QTdw.1*	2B	wmc154a-wmc154b	3.1–3.3	4.25	10.3	Sun et al., 2013
	*QTdw.2*	2B	wPt-0100-wPt-6627	3.1–3.3	−3.50	9.6	Sun et al., 2013
Total fresh weight	*QTfw*	1A	wPt-731490-wPt-6358	2.5–5.7	52.34	14.9	Sun et al., 2013
	*QTfw.1*	1D	wmc432b-wPt-666067	2.5–5.7	−41.08	15.3	Sun et al., 2013
	*QTfw*	2D	wPt-3757-wPt-667054	3.2–5.7	−56.34	10.6	Sun et al., 2013
	*QTfw.1*	4A	srap18-issr23b	4.2–5.9	75.46	11.3	Sun et al., 2013

**TABLE 4:** Detailed description of QTL associated with nitrogen use efficiency in wheat.

Traits	QTL name	Chr	Marker/interval	LOD	ADD	*R*^2^	References
Nitrogen utilization efficiency	*NutE2*	3A	CDO482-CDO482	3.52	–	–	Cormier et al., 2016
	*NutE4*	4A	WPT5172-WPT2780	3.06	–	0.05	Cormier et al., 2016
	*NutE3*	5B	TPT3144-WMC783	3.61	–	0.08	Cormier et al., 2016
	*NutE5*	6D	WPT1519-WPT672044	3.7	–	0.16	Cormier et al., 2016
Nitrogen utilization efficiency to protein	*NutE_Prot12*	1A	GDM33-FBA393	3.93	–	0.14	Cormier et al., 2016
	*NutE_Prot15*	3A	WPT1816-GWM666B	3.25	–	0.01	Cormier et al., 2016
	*NutE_Prot8*	3A	WPT6234-WPT6234	3.75	–	0.02	Cormier et al., 2016
	*NutE_Prot6*	3B	WMM1441-WMM1441	3.23	–	–	Cormier et al., 2016
	*NutE_Prot4*	3D	GDM128-GDM128	3.27	–	–	Cormier et al., 2016
	*NutE_Prot16*	4A	GPW4182-WMC757	3.43	–	0.12	Cormier et al., 2016
	*NutE_Prot14*	5A	WG564-PSB85	3.52	–	0.28	Cormier et al., 2016
	*NutE_Prot17*	5A	GWM241-GWM241	3.46	–	0.02	Cormier et al., 2016
	*NutE_Prot3*	5A	BCD926-GWM186	3.45	–	0.01	Cormier et al., 2016
	*NutE_Prot1*	6A	GPW3251-GPW3251	3.05	–	0.19	Cormier et al., 2016
	*NutE_Prot7_13*	6D	WPT1519-WPT672044	3.62	–	0.2	Cormier et al., 2016
	*NutE_Prot2*	7A	WPT2903-WPT4126	3.1	–	0.04	Cormier et al., 2016
	*NutE_Prot18*	7B	WMC606-WMC323	3.03	–	0.06	Cormier et al., 2016
	*NutE_Prot11*	7D	GPW334-GPW334	4.49	–	0.09	Cormier et al., 2016
Nitrogen amount in the straw	*QNSA*	5A	gwm595	3.3	–	3.33	Laperche et al., 2007a
	*QNSA*	6A	gpw2295	4.3	–	11.8	Laperche et al., 2007a
Nitrogen amount in the straw, straw nitrogen	*QNSA, QNS*	5A	GENO-1	4.6,5.3	–	11.7	Laperche et al., 2007a
Nitrogen concentration at anthesis	*NFA8_7*	3A	TPT1143-GWM638	3.56	–	0.08	Cormier et al., 2016
	*NFA10*	3B	FBB24-FBB24	3	–	0.06	Cormier et al., 2016
	*NFA12*	4A	WMC757-GPW1010	3.08	–	0.08	Cormier et al., 2016
	*NFA5*	4A	GDM141-FBA147	3.09	–	0.04	Cormier et al., 2016
	*NFA2*	5A	WMC524-WMC524	3.02	–	0.06	Cormier et al., 2016
	*NFA11*	5B	WPT2707-WPT2707	4.43	–	0.1	Cormier et al., 2016
	*NFA6*	6B	SHI330-FBB130	3.02	–	0.04	Cormier et al., 2016
	*NFA4*	7D	BARC352-BARC352	3.37	–	0.05	Cormier et al., 2016
Nitrogen concentration at flowering	*%N_FLO8*	5B	WPT8414-CFA2121B	3.8	–	0.26	Cormier et al., 2016
	*%N_FLO10*	6A	WPT1377-WPT730591	3	–	0.09	Cormier et al., 2016
	*%N_FLO5*	6A	PSR312-BARC118	3.09	–	0.01	Cormier et al., 2016
	*%N_FLO6*	6A	GWM169-GPW5125	3.08	–	0.08	Cormier et al., 2016
	*%N_FLO7*	6B	SHI330-FBB130	3.15	–	0.11	Cormier et al., 2016
Nitrogen concentration at maturity	*%N_S19*	1B	KSUF43B-GWM264D	3.97	–	0.11	Cormier et al., 2016
	*%N_S2*	1B	MGL77-WPT2230	6.35	–	0.21	Cormier et al., 2016
	*%N_S4*	1B	KU136-WPT5485	4.17	–	0.08	Cormier et al., 2016
	*%N_S12*	2A	GWM294-BCD1095	3.58	–	0.05	Cormier et al., 2016
	*%N_S20*	2A	CFD55-GWM71D	3.01	–	0.11	Cormier et al., 2016
	*%N_S21*	2A	WMC522-WPT5251	3	–	0.1	Cormier et al., 2016
	*%N_S13*	3A	WMC388C-CDO281	4.4	–	0.05	Cormier et al., 2016
	*%N_S11*	3B	FBB24-FBB24	3.27	–	0.04	Cormier et al., 2016
	*%N_S5*	3B	CFB3023-GPW3092	3.12	–	0.07	Cormier et al., 2016
	*%N_S3*	4A	SHH114-WPT9901	4.17	–	0.04	Cormier et al., 2016
	*%N_S10*	4B	PSP3163-WMC657	3.13	–	0.12	Cormier et al., 2016
	*%N_S7*	4D	GBXG102-BLT101	3.33	–	0.05	Cormier et al., 2016
	*%N_S1*	5B	GDM116-WPT6880	3.15	–	0.05	Cormier et al., 2016
	*%N_S14*	5B	TPT3144-WMC783	4.42	–	0.14	Cormier et al., 2016
	*%N_S15*	5B	SSIB-PSR580	3.82	–	0.03	Cormier et al., 2016
	*%N_S18*	6A	WPT3091-WPT3091	3.35	–	0.13	Cormier et al., 2016
	*%N_S8*	6A	GPW3251-GPW3251	3.3	–	0.03	Cormier et al., 2016
	*%N_S16*	7B	WPT3530-WPT7113	5	–	0.1	Cormier et al., 2016
	*%N_S17*	7B	BARC182-BARC97B	3.45	–	0.09	Cormier et al., 2016
Nitrogen harvest index	*NHI11*	1A	BCD808A-WMC11	3.15	–	0.04	Cormier et al., 2016
	*NHI3*	1B	KSUD14-FBA199	3.54	–	0.11	Cormier et al., 2016
	*NHI7*	1B	KSUF43B-GWM264D	3.68	–	0.08	Cormier et al., 2016
	*NHI1*	2A	WPT9302-WPT9302	3.16	–	0.02	Cormier et al., 2016
	*NHI5*	4B	GPW4075-SHI211	3.27	–	0.04	Cormier et al., 2016
	*NHI6*	5A	GWM595-GWM595	3.55	–	0.07	Cormier et al., 2016
	*NHI8*	5A	DOFA-DOFA	3.56	–	0.03	Cormier et al., 2016
	*NHI9*	5B	WPT8414-WPT0517	3.48	–	0.07	Cormier et al., 2016
	*NHI10*	7A	DUPW226-DUPW226	3.18	–	0.01	Cormier et al., 2016
	*NHI4*	7A	DUPW226-DUPW226	3.17	–	0.05	Cormier et al., 2016
Nitrogen nutrition index	*INN_FLO5*	1B	KSUF43B-GWM264D	3.21	–	0.13	Cormier et al., 2016
	*INN_FLO6*	5B	GWM67-BCD351	3.37	–	0.13	Cormier et al., 2016
	*INN_FLO7*	5B	WPT8414-CFA2121B	3.57	–	0.19	Cormier et al., 2016
	*INN_FLO4*	6A	GWM169-GPW5125	3.48	–	0.07	Cormier et al., 2016
Nitrogen remobilization	*REMN12*	1B	WPT3950-CDO346	3.23	–	0.05	Cormier et al., 2016
	*REMN3*	1B	WPT1972-TPT5249	3.92	–	0.04	Cormier et al., 2016
	*REMN6*	1B	KSUF43B-GWM264D	3.47	–	0.07	Cormier et al., 2016
	*REMN5*	4A	GDM141-FBA147	3.53	–	0.05	Cormier et al., 2016
	*REMN10*	5B	WPT2707-WPT2707	3.7	–	0.08	Cormier et al., 2016
	*REMN9*	6A	WPT5395-WPT4752	3.09	–	0.06	Cormier et al., 2016
	*REMN4*	7A	WMC488-WMC488	3.76	–	0.09	Cormier et al., 2016
	*REMN7*	7A	FBA350-FBA350	3.6	–	0.06	Cormier et al., 2016
	*REMN11*	7B	GPW4471-FBB352	3.05	–	0.06	Cormier et al., 2016
	*REMN8*	7B	WPT3723-WPT5892	3.26	–	0.08	Cormier et al., 2016
	*REMN1*	7D	WPT4555-WPT4555	4.07	–	0.08	Cormier et al., 2016
Nitrogen remobilization efficiency	*EFFREMN8*	1A	WPT-9757-BCD808B	3.44	–	0.06	Cormier et al., 2016
	*EFFREMN10*	1B	GPW4069-WMC500B	3.59	–	0.09	Cormier et al., 2016
	*EFFREMN4*	1B	STM542ACAG-TPT5249	3.39	–	0.07	Cormier et al., 2016
	*EFFREMN11*	3D	GPW4451-GPW4451	3.26	–	0.07	Cormier et al., 2016
	*EFFREMN13*	3D	GPW7053-WPT742732	3.3	–	0.05	Cormier et al., 2016
	*EFFREMN3*	4A	WPT3638-WPT4660	3	–	0.08	Cormier et al., 2016
	*EFFREMN2*	5A	PSY-GPW3049	4.37	–	0.08	Cormier et al., 2016
	*EFFREMN7*	6A	GPW3251-GPW3251	3.24	–	0.07	Cormier et al., 2016
	*EFFREMN12*	7B	BARC97B-KSUE18B	3	–	0.06	Cormier et al., 2016
	*EFFREMN5*	7B	WPT9813-WPT1196	3.89	–	0.09	Cormier et al., 2016
	*EFFREMN9*	7B	WPT8890-WPT4230	3.32	–	0.08	Cormier et al., 2016
Nitrogen uptake	*Qnup-1*	2D	Xgwm539-P4233-175	2.08	−5.00	6	An et al., 2006
	*Qnup-2*	4B	Xgwm495-Xgwm149	2.26	4.00	5.2	An et al., 2006
	*Qnup-3*	6A	WMC179.1-WMC256	7.25	9.00	21.9	An et al., 2006
	*Qnup-4*	6B	P3454-165-P3516-205	4.26	−6.00	10.9	An et al., 2006
	*Qnup-5*	2B	WMC272-Xgwm319	3.04	−5.00	8.3	An et al., 2006
	*Qnup-6*	4A	WMC89-WMC420	2.14	4.00	6.3	An et al., 2006
	*Qnup-7*	5B	WMC363-WMC376	4.14	6.00	12.4	An et al., 2006
	*Qnup-8*	6A	WMC179.1-WMC256	2.62	5.00	8.3	An et al., 2006
	*Qnup-9*	7D	Xgdm88-WMC463	2.69	6.00	10.1	An et al., 2006
	*Qnup-10*	3B	P2076-147-Xgwm108	2.57	−6.00	7	An et al., 2006
	*Qnup-11*	5A	Xgwm415-Xgwm304	3.3	−7.00	8.6	An et al., 2006
	*Qnup-12*	5A	Xgwm595-WMC410	5.74	9.00	15.9	An et al., 2006
	*Qnup-13*	7B	Xgwm400-P6401-238	2.33	6.00	6.8	An et al., 2006
	*Qnup-14*	1B	WMC156-P3446-183	2.4	−6.00	6.4	An et al., 2006
	*Qnup-15*	2D	Xgwm157-Xgwm539	4.31	−9.00	14	An et al., 2006
	*Qnup-16*	3B	Xgwm108-WMC291	2.67	−7.00	7.3	An et al., 2006
	*Qnup-17*	4B	Xgwm495-Xgwm149	4.24	9.00	14.1	An et al., 2006
Nitrogen use efficiency	*NUE8*	1A	GDM33-FBA393	3.58	–	0.12	Cormier et al., 2016
	*NUE10*	3A	WPT1816-GWM666B	4.26	–	0.01	Cormier et al., 2016
	*NUE6*	3A	WPT6234-WPT6234	4.78	–	0.03	Cormier et al., 2016
	*NUE2*	3B	CFB3440-CFB3440	3.13	–	0.09	Cormier et al., 2016
Nitrogen utilization efficiency for grain yield	*QNUtEGY*	4D	Xgdm14.2-Xcfd71	–	−0.77	8.8	Xu et al., 2013
	*NUE5*	3B	WMM1441-WMM1441	3.33	–	0.01	Cormier et al., 2016
	*NUE3*	3D	GDM128-GDM128	3.29	–	–	Cormier et al., 2016
	*NUE11*	4A	GPW4182-WMC757	3.15	–	0.11	Cormier et al., 2016
	*NUE14*	5A	GWM241-GWM241	3.35	–	0.02	Cormier et al., 2016
	*NUE12*	6D	WPT1519-WPT672044	3.07	–	0.18	Cormier et al., 2016
	*NUE1*	7A	BARC174-GWM631	4.27	–	0.12	Cormier et al., 2016
	*NUE13*	7A	BARC222-WPT8897	3.03	–	0.13	Cormier et al., 2016
	*NUE7*	7D	GPW334-GPW334	3.58	–	0.06	Cormier et al., 2016
	*qNUE*	2D	Xgwm539-P4233-175	2.08	−5.00	6	An et al., 2006
	*qNUE*	4B	Xgwm495-Xgwm149	2.26	4.00	5.2	An et al., 2006
	*qNUE*	6A	WMC179.1-WMC256	7.25	9.00	21.9	An et al., 2006
	*qNUE*	6B	P3454-165-P3516-205	4.26	−6.00	10.9	An et al., 2006
	*qNUE*	2B	WMC272-Xgwm319	3.04	−5.00	8.3	An et al., 2006
	*qNUE*	4A	WMC89-WMC420	2.14	4.00	6.3	An et al., 2006
	*qNUE*	5B	WMC363-WMC376	4.14	6.00	12.4	An et al., 2006
	*qNUE*	6A	WMC179.1-WMC256	2.62	5.00	8.3	An et al., 2006
	*qNUE*	7D	Xgdm88-WMC463	2.69	6.00	10.1	An et al., 2006
	*qNUE*	3B	P2076-147-Xgwm108	2.57	−6.00	7	An et al., 2006
	*qNUE*	5A	Xgwm415-Xgwm304	3.3	−7.00	8.6	An et al., 2006
	*qNUE*	5A	Xgwm595-WMC410	5.74	9.00	15.9	An et al., 2006
	*qNUE*	7B	Xgwm400-P6401-238	2.33	6.00	6.8	An et al., 2006
	*qNUE*	1B	WMC156-P3446-183	2.4	−6.00	6.4	An et al., 2006
	*qNUE*	2D	Xgwm157-Xgwm539	4.31	−9.00	14	An et al., 2006
	*qNUE*	3B	Xgwm108-WMC291	2.67	−7.00	7.3	An et al., 2006
	*qNUE*	4B	Xgwm495-Xgwm149	4.24	9.00	14.1	An et al., 2006
	*QRfw.2*	4A	srap7b-srap7c	24.6–30.0	87.85	26.5	Sun et al., 2013
	*QTfw.2*	4A	srap7b-srap7c	28.9–35.5	218.02	10	Sun et al., 2013
	*QRsfw.2*	4A	srap7b-srap7c	24.6–30.0	0.48	11.2	Sun et al., 2013
	*QTdw*	4A	srap7b-srap7c	32.2–35.4	18.60	10	Sun et al., 2013
	*QRsdw*	4A	srap7b-srap7c	3.9–4.9	0.03	11.7	Sun et al., 2013
	*QSfw*	4B	wPt7569-wPt3991	3.1–3.6	−38.24	10.1	Sun et al., 2013
	*QTfw*	4B	wPt7569-wPt3991	3.2–4.7	−50.55	11.4	Sun et al., 2013
	*QSfw.1*	5B	wPt-0103-wPt-6052	4.0–6.1	27.95	12.8	Sun et al., 2013
	*QTfw*	5B	wPt-0103-wPt-6052	3.4–4.7	42.95	11.3	Sun et al., 2013
	*QRdw.1*	5B	wPt-0103-wPt-6052	3.8–4.6	1.06	11.5	Sun et al., 2013
	*QTdw*	5B	wPt-0103-wPt-6052	3.5–4.4	4.47	10.3	Sun et al., 2013
	*QTfw*	5D	swes555b-swes558a	3.0–3.9	32.92	8.2	Sun et al., 2013
	*QSdw*	5D	swes555b-swes558a	3.3–4.0	3.08	8.7	Sun et al., 2013
	*QTfw.1*	6B	swes1-wPt-5176	3.2–5.8	33.29	12.7	Sun et al., 2013
	*QSdw.1*	6B	swes1-wPt-5176	3.3–4.9	2.71	11.6	Sun et al., 2013
	*QRsdw.1*	6B	swes1-wPt-5176	3.2–4.7	−0.01	8.2	Sun et al., 2013
	*QRfw*	7A	barc121-ubc811a	3.4–4.7	16.67	10.5	Sun et al., 2013
	*QSfw*	7A	barc121-ubc811a	4.0–6.1	−36.02	14.3	Sun et al., 2013
	*QTfw*	7A	barc121-ubc811a	3.5–4.4	−51.72	13.1	Sun et al., 2013
	*QRsfw.3*	7A	barc121-ubc811a	3.0–3.2	0.02	9.6	Sun et al., 2013
	*QSdw.1*	7A	barc121-ubc811a	3.7–4.1	−5.12	13.2	Sun et al., 2013
	*QTdw.2*	7A	barc121-ubc811a	3.5–3.5	−7.00	11.5	Sun et al., 2013
	*QRsdw.1*	7A	barc121-ubc811a	3.6–5.6	0.02	11.1	Sun et al., 2013
	*QRfw*	7B	wPt-0194-wPt-2305	3.2–4.7	−41.74	21.6	Sun et al., 2013
	*QTfw*	7B	wPt-0194-wPt-2305	3.2–5.8	−110.31	14.4	Sun et al., 2013
	*QRsfw.2*	7B	wPt-0194-wPt-2305	3.7–4.1	−0.12	16.3	Sun et al., 2013
	*QRdw*	7B	wPt-0194-wPt-2305	3.7–4.1	−4.61	27.7	Sun et al., 2013
	*NupEFlo6_5*	3A	TPT1143-GWM638	3.96	–	0.08	Cormier et al., 2016
	*NupEFlo3*	4A	GDM141-FBA147	3.65	–	0.05	Cormier et al., 2016
	*QSdw*	7B	wPt-0194-wPt-2305	3.4–4.7	−8.06	11.1	Sun et al., 2013
Nitrogen use efficiency at anthesis	*NupEFlo2*	2A	WMC181C-WPT8326	3.28	–	0.07	Cormier et al., 2016
	*NupEFlo8*	5B	WPT2707-WPT2707	5.47	–	0.13	Cormier et al., 2016
	*NupEFlo4*	6B	SHI330-FBB130	3.22	–	0.04	Cormier et al., 2016
	*NupEFlo9*	7A	WMC488-WMC488	3.08	–	0.05	Cormier et al., 2016
Nitrogen use efficiency at maturity	*NupEMat5*	1B	DUPW214B-WMC430	3.42	–	0.02	Cormier et al., 2016
	*NupEMat6*	1B	WPT0697-BCD1124	3.2	–	0.05	Cormier et al., 2016
	*NupEMat8*	1D	WPT8854-GPW300	3.57	–	0.09	Cormier et al., 2016
	*NupEMat3*	2A	GWM400-MRGA2	3.82	–	0.09	Cormier et al., 2016
	*NupEMat4*	4A	GWM397-GPW7020	3.06	–	0.06	Cormier et al., 2016
	*NupEMat1*	5A	TPT9702-WPT0605	3.44	–	0.08	Cormier et al., 2016
	*NupEMat11*	5A	ABG366-ABG366	3.01	–	0.08	Cormier et al., 2016
	*NupEMat10*	5B	FBA342-GBXG198	3.08	–	0.02	Cormier et al., 2016
	*NupEMat9*	6A	WPT0696-WPT9474	3.3	–	0.09	Cormier et al., 2016
	*NupEMat2*	7A	WMC488-WPT2083	3.28	–	0.03	Cormier et al., 2016
Nitrogen use efficiency to protein	*NUE_Prot3*	3A	CDO482-CDO482	3.36	–	−0.01	Cormier et al., 2016
	*NUE_Prot7*	3B	WMC540-WMC540	3.07	–	0.12	Cormier et al., 2016
	*NUE_Prot9*	4A	WPT5172-WPT2780	3.14	–	0.04	Cormier et al., 2016
	*NUE_Prot5*	5A	WG564-PSB85	4.22	–	0.23	Cormier et al., 2016
	*NUE_Prot2*	5B	WPT6726-DUPW395	3.18	–	0.26	Cormier et al., 2016
	*NUE_Prot6*	5B	WPT0517-GDM116	3.05	–	0.04	Cormier et al., 2016
	*NUE_Prot8*	6D	WPT1519-WPT672044	4.48	–	0.2	Cormier et al., 2016
	*NUE_Prot4*	7B	BE499017-WMC546C	3.26	–	0.12	Cormier et al., 2016
Straw nitrogen	*QNS*	2A	gwm497d	3.5	–	7	Laperche et al., 2007b
	*QNS*	5D	gwm639c	3.9	–	7.5	Laperche et al., 2007a
	*QNS*	7A	gwm635	3.1	–	9.2	Laperche et al., 2007a
	*NSA1*	1B	WPT1399-WPT5485	3.16	–	0.05	Cormier et al., 2016
	*NSA4*	2A	BQ161439-FBB353	3.73	–	0.02	Cormier et al., 2016
	*NSA6*	3B	WPT1336-WPT1741	3.42	–	0.04	Cormier et al., 2016
	*NSA8*	3B	FBB24-FBB24	3.36	–	0.08	Cormier et al., 2016
	*NSA14*	3D	GPW7053-WPT742732	3.38	–	0.02	Cormier et al., 2016
	*NSA2*	4A	SHH114-FBB154	3.64	–	0.04	Cormier et al., 2016
	*NSA10*	5A	GWM595-GWM595	3.05	–	0.08	Cormier et al., 2016
	*NSA9*	5B	TPT3144-WMC783	3.56	–	0.12	Cormier et al., 2016
	*NSA15*	7A	DUPW226-DUPW226	3.54	–	0.03	Cormier et al., 2016
	*NSA11*	7B	GPW4471-FBB352	3.15	–	0.11	Cormier et al., 2016
Straw nitrogen uptake	*QSnup*	5A.1	Xgwm328-Xlhq87	–	0.004	8.9	Xu et al., 2013
Total nitrogen per area	*NTA3*	1B	WPT0697-BCD1124	3.18	–	0.04	Cormier et al., 2016
	*NTA7*	1D	WPT8854-GPW300	4.32	–	0.11	Cormier et al., 2016
	*NTA2*	2A	GWM400-MRGA2	3.84	–	0.09	Cormier et al., 2016
	*NTA6*	5A	ABG366-ABG366	3.45	–	0.1	Cormier et al., 2016
	*NTA8*	6A	WPT0696-WPT9474	3.02	–	0.09	Cormier et al., 2016
	*NTA9*	6A	GWM427-TPT4178	3.19	–	0.05	Cormier et al., 2016
	*NTA1*	7A	WMC488-WPT2083	3.33	–	0.03	Cormier et al., 2016

Significant variability and marker-trait associations in genome-wide association studies for nitrogen uptake and use efficiency have been reported (Barraclough et al., 2010; Liu Z. et al., 2016; Monostori et al., 2017). Genome-wide association studies were conducted exploiting the phenotypic variability of the nested synthetic wheat introgression libraries developed at Punjab Agricultural University, Ludhiana (India). Several marker-trait associations associated with root and plant morphological traits, grain yield, and yield-related traits were identified (data unpublished). QTL associated with root-traits and nutrient-uptake (Sandhu et al., 2015, Sandhu et al., 2016) in rice have been reported. Several genetic regions associated with nutrient uptake have been detected in rice (Wissuwa et al., 1998; Ming et al., 2000), wheat (Su et al., 2006, 2009), maize (Zhu et al., 2005), common bean (Liao et al., 2004; Yan et al., 2004), and soybean (Li et al., 2005; Liang et al., 2010). Comparative mapping involving other cereal crops such as rice aims to identify highly conserved sequences, new genes, and regulatory elements to link genomes, genes, proteins, and traits controlling traits of interest across different species and genera. These inter-genome relational patterns can lead to new hypotheses, knowledge, and predictions about the related species.

### Genes Related to NUE

**FIGURE 6:**
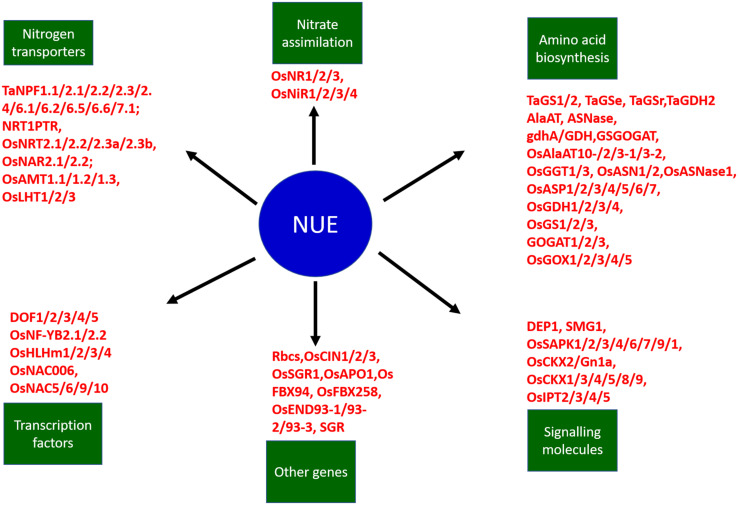
The schematic representation of the network of nitrogen transporters, genes, transcription factors, and signaling molecules involved in nitrogen use efficiency in cereal crops such as wheat and rice. NUE improvement involves multiple interconnected factors such as transporters, signal molecules, amino acid biosynthesis, nitrate assimilation, transcription factors, and other genes. The transporters and nitrate assimilation genes are particularly involved in nitrogen uptake, amino acid biosynthesis genes are involved in nitrogen utilization, and the signaling molecules, transcription factors, and other genes have passive roles in both nitrogen uptake and nitrogen utilization. Ta represents *Triticum aestivum* and Os represents *Oryza sativa.*

Multiple sets of genes in crop plants are known to regulate the mechanisms associated with NUE such as nitrogen absorption, accumulation, and remobilization. Genes regulating NUE among different cereal crops such as wheat and rice are broadly divided into six categories including, transporters, signal molecules, amino acid biosynthesis, nitrate assimilation, transcription factors, and other genes (Figure 6). The detailed description of network genes associated with nitrogen use efficiency in rice crops is presented in Table 5 and in wheat in Table 6. Among these categories, transporters and nitrate assimilation genes are particularly involved in nitrogen uptake, and amino acid biosynthesis genes are involved in nitrogen utilization, whereas signaling molecules, transcription factors, and other genes have a passive role in both nitrogen uptake and nitrogen utilization (Zhou et al., 2009).

**TABLE 5:** Detailed description of network genes associated with nitrogen use efficiency in rice crop.

Category	Gene	Chr	Location	Locus name	Gene family	Phenotypic description	References
** Transporters**	*OsNRT2.1*	2	655,324–657,243	*LOC_Os02g02170*	Nitrate transporter 2 (high affinity)	High-affinity nitrate transporter, nitrate uptake, nitrate transporter	Han et al., 2016
	*OsNRT2.2*	2	667,264–669,053	*LOC_Os02g02190*	Nitrate transporter 2 (high affinity)	High-affinity nitrate transporter, nitrate uptake, nitrate transport	Han et al., 2016
	*OsNRT2.3a*	1	29,188,850–29,190,936	*LOC_Os01g50820*	Nitrate transporter 2 (high affinity)	Nitrate transporter, nitrate transporter	Han et al., 2016
	*OsNRT2.3b*	1	29,188,850–29,190,936	*LOC_Os01g50820*	Nitrate transporter 2 (high affinity)	Nitrate transporter, nitrate transporter	Han et al., 2016
	*OsNAR2.1*	2	23,121,133–23,123,149	*LOC_Os02g38230*	NRT2 partner protein (NAR2)	Partner protein of NRT2, activator for NRT2, high-affinity nitrate transporter	Han et al., 2016
	*OsNAR2.2*	4	24,018,298–24,019,456	*LOC_Os04g40410*	NRT2 partner protein (NAR2)	Transporter, high-affinity nitrate, Nar2 domain containing protein	Han et al., 2016
	*OsAMT1.1*	4	25,500,515–25,502,557	*LOC_Os04g43070*	Ammonium transporter	Ammonium transporter, ammonium uptake, ammonium transport	Han et al., 2016
	*OsAMT1.2*	2	24,683,709–24,685,205	*LOC_Os02g40710*	Ammonium transporter	Similar to ammonium transporter Amt1;2 (fragment)	Han et al., 2016
	*OsAMT1.3*	2	24,690,884–24,692,736	*LOC_Os02g40730*	Ammonium transporter	Ammonium transporter, ammonium uptake	Han et al., 2016
	*OsLHT1*	12	7,997,383–8,000,365	*LOC_Os12g14100*	Lysine histidine transporter	Similar to lysine and histidine specific transporter	Han et al., 2016
	*OsLHT2*	12	561,754–1,566,957	*LOC_Os08g03350*	Lysine histidine transporter	Amino acid transporter, transmembrane family protein	Han et al., 2016
	*OsLHT3*	5	8,427,409–8,429,533	*LOC_Os05g14820*	Lysine histidine transporter	Similar to histidine amino acid transporter	Han et al., 2016
** Amino acid biosynthesis**	*AlaAT*	10	12,968,039–12,974,099	*Os10g0390500*	Alanine aminotransferase	Alanine aminotransferase, starch synthesis in developing seed	Shrawat et al., 2008
	*ASNase*	3	22,255,220–22,259,151	*Os03g0597600*	Asparaginase	Similar to L-asparaginase (EC 3.5.1.1) (L-asparagine amidohydrolase)	Zhou et al., 2009
	*gdhA/GDH*	2	31,541,674–31,545,959	*ONIVA02G36440*	NADP-dependent glutamate dehydrogenase	Several fold higher levels of free amino acids including glutamate	Abiko et al., 2010
	*GS*	3	12,021,878–12,022,283	*Os03g0328400*	Glutamine synthetase	Similar to apyrase GS50 (fragment), NUE increased under high N condition	Brauer et al., 2011
	*GOGAT*	1	28,091,236–28,091,512	*Os01g0681900*	Glutamate synthase	Non-protein coding transcript	Tamura et al., 2011
	*OsAlaAT10-2*	10	12,977,443–12,982,476	*Os10g0390600*	Glutamic-pyruvate transaminase (alanine aminotransferase; GPT)	Similar to alanine aminotransferase 2 (EC 2.6.1.2) (GPT) (glutamic–pyruvic transaminase 2) (glutamic–alanine transaminase 2) (ALAAT-2)	Han et al., 2016
	*OsAlaAT2*	9	15,930,510–15,936,552	*Os09g0433900*	Glutamic-pyruvate transaminase (alanine aminotransferase; GPT)	Similar to alanine aminotransferase 2 (EC 2.6.1.2) (GPT) (glutamic–pyruvic transaminase 2) (Glutamic–alanine transaminase 2) (ALAAT-2)	Han et al., 2016
	*OsAlaAT3-1*	7	450,801–451,136	*Os07g0108350*	Glutamic-pyruvate transaminase (alanine aminotransferase; GPT)	Hypothetical conserved gene	Han et al., 2016
	*OsAlaAT3-2*	7	25,492,989–25,495,303	*Os07g0617800*	Glutamic-pyruvate transaminase (alanine aminotransferase; GPT)	Similar to alanine aminotransferase	Han et al., 2016
	*OsGGT1*	5	23,356,169–23,360,022	*Os05g0475400*	Glutamate glyoxylate aminotransferase (GGT)	Similar to alanine: glyoxylate aminotransferase-like protein (fragment)	Han et al., 2016
	*OsGGT3*	3	12,556,849–12,559,702	*Os03g0338000*	Glutamate glyoxylate aminotransferase (GGT)	Similar to alanine-glyoxylate aminotransferase 2	Han et al., 2016
	*OsASN1*	3	10,120,289–10,124,384	*Os03g0291500*	Asparagine synthetase	Asparagine synthetase, biosynthesis of asparagine following the supply of ammonia	Han et al., 2016
	*OsASN2*	6	8,758,936–8,765,680	*Os06g0265000*	Asparagine synthetase	Asparagine synthetase, long-distance transport of asparagine	Han et al., 2016
	*OsASNase1*	4	27,494,477–27,497,968	*Os04g0549300*	Asparaginase	Similar to GA protein (fragment)	Han et al., 2016
	*OsASP1*	2	33,942,024–33,946,388	*Os02g0797500*	Aspartate aminotransferase	Similar to plastidic aspartate aminotransferase	Han et al., 2016
	*OsASP2*	6	20,727,293–20,731,768	*Os06g0548000*	Aspartate aminotransferase	Aspartate aminotransferase (EC 2.6.1.1)	Han et al., 2016
	*OsASP3*	2	7,706,619–7,710,902	*Os02g0236000*	Aspartate aminotransferase	Similar to aspartate aminotransferase (EC 2.6.1.1) (fragment)	Han et al., 2016
	*OsASP4*	1	31,998,877–32,003,690	*Os01g0760600*	Aspartate aminotransferase	Aspartate aminotransferase, cytoplasmic (EC 2.6.1.1) (transaminase A)	Han et al., 2016
	*OsASP5*	1	37,779,512–37,782,837	*Os01g0871300*	Aspartate aminotransferase	Pyridoxal phosphate-dependent transferase, major region domain containing protein	Han et al., 2016
	*OsASP6*	10	18,311,854–18,314,316	*Os10g0484700*	Aspartate aminotransferase	Pyridoxal phosphate-dependent transferase, major region, subdomain 1 domain containing protein	Han et al., 2016
	*OsASP7*	9	17,024,575–17,028,546	*Os09g0453800*	Aspartate aminotransferase	1-aminocyclopropane-1-carboxylate synthase family protein	Han et al., 2016
	*OsGDH1*	3	33,037,377–33,042,153	*Os03g0794500*	Glutamate dehydrogenase NAD(P)H	Similar to glutamate dehydrogenase (EC 1.4.1.3) (GDH)	Han et al., 2016
	*OsGDH2*	4	26,019,972–26,025,400	*Os04g0543900*	Glutamate dehydrogenase NAD(P)H	Glutamate dehydrogenase 2, mitochondrial	Han et al., 2016
	*OsGDH3*	2	26,239,683–26,243,529	*Os02g0650900*	Glutamate dehydrogenase NAD(P)H	Similar to glutamate dehydrogenase 2 (EC 1.4.1.3) (GDH 2)	Han et al., 2016
	*OsGDH4*	1	21,118,894–21,124,700	*Os01g0558200*	Glutamate dehydrogenase NAD(P)H	Glutamate/phenylalanine/leucine/valine dehydrogenase domain containing protein	Han et al., 2016
	*OsGS1*	2	30,674,004–30,679,435	*Os02g0735200*	Glutamine synthetase	Glutamine synthetase shoot isozyme (EC 6.3.1.2) (glutamate–ammonia ligase) (clone lambda-GS28)	Han et al., 2016
	*OsGS2*	3	6,457,915–6,462,146	*Os03g0223400*	Glutamine synthetase	Cytosolic glutamine synthetase, ammonium assimilation	Han et al., 2016
	*OsGS3*	3	28,822,424–28,826,321	*Os03g0712800*	Glutamine synthetase	Cytosolic glutamine synthetase	Han et al., 2016
	*GOGAT1*	1	28,098,847–28,102,930	*Os01g0682001*	Glutamate synthase (NADPH/ferredoxin)	Similar to NADH dependent glutamate synthase	Han et al., 2016
	*GOGAT2*	7	27,723,089–27,738,212	*Os07g0658400*	Glutamate synthase (NADPH/ferredoxin)	Ferredoxin-dependent glutamate synthase, leaf senescence and nitrogen remobilization	Han et al., 2016
	*GOGAT3*	5	27,631,211–27,636,450	*Os05g0555600*	Glutamate synthase (NADPH/ferredoxin)	Similar to glutamate synthase [NADH], amyloplastic	Han et al., 2016
	*OsGOX1*	3	32,628,790–32,632,431	*Os03g0786100*	Glycolate oxidase (GOX)	Glycolate oxidase, photorespiratory enzyme, strong regulation over photosynthesis, feedback inhibition on Rubisco activity	Han et al., 2016
	*OsGOX2*	4	31,688,721–31,692,502	*Os04g0623500*	Glycolate oxidase (GOX)	Similar to H0215F08.7 protein	Han et al., 2016
	*OsGOX3*	4	31,693,183–31,696,603	*Os04g0623600*	Glycolate oxidase (GOX)	Similar to peroxisomal (S)-2-hydroxy-acid oxidase GLO2	Han et al., 2016
	*OsGOX4*	7	2,797,691–2,801,343	*Os07g0152900*	Glycolate oxidase (GOX)	Similar to glycolate oxidase (EC 1.1.3.15) (fragment)	Han et al., 2016
	*OsGOX5*	7	25,408,400–25,413,093	*Os07g0616500*	Glycolate oxidase (GOX)	Similar to (S)-2-hydroxy-acid oxidase, peroxisomal (EC 1.1.3.15) (glycolate oxidase) (GOX) (short-chain alpha-hydroxy acid oxidase)	Han et al., 2016
**Nitrate assimilation**	*OsNR1*	8	23,051,707–23,055,631	*Os08g0468700*	Nitrate reductase	Similar to nitrate reductase	Han et al., 2016
	*OsNR2*	2	32,513,749–32,517,155	*Os02g0770800*	Nitrate reductase	Similar to nitrate reductase [NAD(P)H] (EC 1.7.1.2)	Han et al., 2016
	*OsNR3*	8	23,033,230–23,038,585	*Os08g0468100*	Nitrate reductase	Similar to nitrate reductase	Han et al., 2016
	*OsNiR1*	1	14,446,913–14,453,454	*Os01g0357100*	Ferredoxin-nitrite reductase	Ferredoxin-nitrite reductase, nitrate reduction (assimilation), determination of regeneration ability	Han et al., 2016
	*OsNiR2*	1	14,462,311–14,462,787	*Os01g0357500*	Ferredoxin-nitrite reductase	Similar to ferredoxin-nitrite reductase	Han et al., 2016
	*OsNiR3*	2	32,254,101–32,257,127	*Os02g0765900*	Ferredoxin-nitrite reductase	Similar to ferredoxin-nitrite reductase	Han et al., 2016
	*OsNiR4*	5	24,777,441–24,782,045	*Os05g0503300*	Ferredoxin-nitrite reductase	Similar to sulfite reductase (fragment)	Han et al., 2016
**Signaling Molecules**	*DEP1*	9	16,411,151 - 16,415,851	*Os09g0441900*	G-protein γ subunit	Cell signaling	Sun et al., 2014
	*SMG1*	2	33,442,070–33,443,948	*Os02g0787300*	Mitogen-activate kinase kinase	Mitogen-activated protein kinase kinase 4, defense response, cell proliferation, grain growth	Duan et al., 2014
	*OsSAPK1*	3	15,628,109–15,632,425	*Os03g0390200*	Sucrose non-fermenting-1 related kinases (SnRK)	Serine/threonine protein kinase, hyperosmotic stress response	Han et al., 2016
	*OsSAPK2*	7	25,717,837–25,722,009	*Os07g0622000*	Sucrose non-fermenting-1 related kinases (SnRK)	Serine/threonine protein kinase, hyperosmotic stress response, abscisic acid (ABA)-dependent gene regulation	Han et al., 2016
	*OsSAPK3*	3	31,652,794–31,658,094	*Os03g0764800*	Sucrose non-fermenting-1 related kinases (SnRK)	Serine/threonine protein kinase, abscisic acid (ABA)-activated protein kinase, hyperosmotic stress response, ABA signal transduction	Han et al., 2016
	*OsSAPK4*	1	37,710,241–37,714,835	*Os01g0869900*	Sucrose non-fermenting-1 related kinases (SnRK)	Serine/threonine protein kinase, hyperosmotic stress response	Han et al., 2016
	*OsSAPK6*	10	22,294,896–22,297,645	*Os10g0564500*	Sucrose non-fermenting-1 related kinases (SnRK)	Serine/threonine protein kinase, hyperosmotic stress response	Han et al., 2016
	*OsSAPK7*	4	21,414,495–21,419,953	*Os04g0432000*	Sucrose non-fermenting-1 related kinases (SnRK)	Similar to serine/threonine-protein kinase SAPK7	Han et al., 2016
	*OsSAPK10*	3	23,068,746–23,071,156	*Os03g0610900*	Sucrose non-fermenting-1 related kinases (SnRK)	Serine/threonine protein kinase, abscisic acid (ABA)-activated protein kinase, hyperosmotic stress response, ABA signal transduction	Han et al., 2016
	*OsSAPK9*	12	24,459,198–24,462,001	*Os12g0586100*	Sucrose non-fermenting-1 related kinases (SnRK)	Serine/threonine protein kinase, abscisic acid (ABA)-activated protein kinase, hyperosmotic stress response, ABA signal transduction	Han et al., 2016
	*OsCKX2/Gn1a*	1	5,270,449–5,275,585	*Os01g0197700*	Cytokinin oxidase/dehydrogenase (CKX)	Cytokinin oxidase/dehydrogenase, regulation of grain production	Han et al., 2016
	*OsCKX5*	1	32,787,636–32,793,599	*Os01g0775400*	Cytokinin oxidase/dehydrogenase (CKX)	Similar to cytokinin dehydrogenase 5 precursors (EC 1.5.99.12) (cytokinin oxidase 5) (CKO5) (AtCKX5) (AtCKX6)	Han et al., 2016
	*OsCKX4*	1	41,300,203–41,302,983	*Os01g0940000*	Cytokinin oxidase/dehydrogenase (CKX)	Cytokinin oxidase/dehydrogenase, crown root formation	Han et al., 2016
	*OsCKX3*	10	18,270,328–18,274,523	*Os10g0483500*	Cytokinin oxidase/dehydrogenase (CKX)	FAD linked oxidase, N-terminal domain containing protein	Han et al., 2016
	*OsCKX1*	1	4,697,238–4,699,036	*Os01g0187600*	Cytokinin oxidase/dehydrogenase (CKX)	Similar to cytokinin dehydrogenase 1	Han et al., 2016
	*OsCKX8*	4	18,032,481–18,035,180	*Os05g0374200*	Cytokinin oxidase/dehydrogenase (CKX)	Similar to cytokinin dehydrogenase 2	Han et al., 2016
	*OsCKX9*	5	18,031,941–18,035,720	*LOC_Os02g12780*	Cytokinin oxidase/dehydrogenase (CKX)	Similar to cytokinin dehydrogenase 2	Han et al., 2016
	*OsIPT2*	3	13,796,252–13,799,655	*Os03g0356900*	Cytokinin biosynthesis (IPT)	tRNA isopentenyl transferase family protein	Han et al., 2016
	*OsIPT3*	5	14,261,484–14,262,509	*Os05g0311801*	Cytokinin biosynthesis (IPT)	Similar to isopentenyl transferase IPT7	Han et al., 2016
	*OsIPT4*	3	33,905,826–33,907,496	*Os03g0810100*	Cytokinin biosynthesis (IPT)	Similar to TRNA isopentenyl transferase-like protein (adenylate isopentenyl transferase) (EC 2.5.1.27)	Han et al., 2016
	*OsIPT5*	7	6,089,992–6,091,035	*Os07g0211700*	Cytokinin biosynthesis (IPT)	Similar to isopentenyl transferase IPT4	Han et al., 2016
**Transcription factors**	*DOF*	3	9,359,575–9,359,994	*Os03g0276300*	DNA-binding one zinc finger	Similar to DOF domain, zinc finger family protein, expressed, increased growth, N assimilation, and enhanced grain production	Li et al., 2013
	*DOF1*	8	24,232,676–24,233,936	*Os08g0490100*	DNA-binding one zinc finger (DOF)	Similar to PBF protein	Han et al., 2016
	*DOF2*	12	24,724,344–24,724,451	*Os12g0590700*	DNA-binding one zinc finger (DOF)	Similar to DOF domain, zinc finger family protein	Han et al., 2016
	*DOF3*	3	31,662,332–31,663,957	*Os03g0764900*	DNA-binding one zinc finger (DOF)	Similar to Zn finger protein (fragment)	Han et al., 2016
	*DOF4*	9	18,234,972–18,235,872	*Os09g0475800*	DNA-binding one zinc finger (DOF)	Transcriptional activator, regulation of the C4 photosynthesis gene, OsC4PPD	Han et al., 2016
	*DOF5*	5	658,112–660,002	*Os05g0112200*	DNA-binding one zinc finger (DOF)	Similar to Zn finger protein (fragment)	Han et al., 2016
	*OsNF-YB2.1*	5	22,770,094–22,774,082	*Os05g0463800*	Nuclear factor Y (NFY)	Similar to nuclear transcription factor Y subunit B-3	Han et al., 2016
	*OsNF-YB2.2*	1	35,756,352–35,758,663	*Os01g0834400*	Nuclear factor Y (NFY)	Similar to HAP3	Han et al., 2016
	*OsHLHm1*	3	6,826,703–6,832,274	*Os03g0229100*	bHLH transcriptional factor	Similar to helix-loop-helix DNA-binding domain containing protein	Han et al., 2016
	*OsHLHm2*	3	29,516,834–29,519,083	*Os03g0725800*	bHLH transcriptional factor	Helix-loop-helix DNA-binding domain containing protein	Han et al., 2016
	*OsHLHm3*	10	345,785–347,108	*Os10g0104300*	bHLH transcriptional factor	Helix-loop-helix DNA-binding domain containing protein	Han et al., 2016
	*OsHLHm4*	12	27,088,697–27,091,800	*Os12g0632600*	bHLH transcriptional factor	Similar to helix-loop-helix DNA-binding domain containing protein	Han et al., 2016
	*OsNAC006*	3	23,734,580–23,736,562	*Os03g0624600*	NAM, ATAF1,2, and CUC2 (NAC)	No apical meristem (NAM) protein domain containing protein	Han et al., 2016
	*OsNAC5*	8	5,846,866–5,850,647	*Os08g0200600*	NAM, ATAF1,2, and CUC2 (NAC)	NAC transcription factor, negative regulation of drought tolerance	Han et al., 2016
	*OsNAC6*	6	28,037,569–28,041,881	*Os06g0675600*	NAM, ATAF1,2, and CUC2 (NAC)	NAC transcription factor, positive regulator of heading and senescence during the reproductive phase	Han et al., 2016
	*OsNAC9/SNAC1*	3	34,166,100–34,167,521	*Os03g0815100*	NAM, ATAF1,2, and CUC2 (NAC)	Similar to OsNAC6 protein	Han et al., 2016
	*OsNAC10*	11	1,233,932–1,235,977	*Os11g0126900*	NAM, ATAF1,2, and CUC2 (NAC)	NAC-domain protein, drought tolerance	Han et al., 2016
**Other Genes**	*Rbcs*	2A	171,076,784–171,079,172	*TraesCS2A02G198700*	Rubisco gene	Gene for the small subunit of the chloroplast photosynthetic enzyme rib- ulose-1,5-bisphosphate carboxylase/oxygenase (Rubisco)	Laperche et al., 2006
	*OsCIN1*	2	19,682,544–19,687,163	*Os02g0534400*	Cell wall invertase	Cell wall invertase (EC 3.2.1.26)	Han et al., 2016
	*GIF1/OsCIN2*	4	20,422,171–20,426,921	*Os04g0413500*	Cell wall invertase	Cell-wall invertase, carbon partitioning during early grain filling	Han et al., 2016
	*OsCIN3*	4	20,412,316–20,415,240	*Os04g0413200*	Cell wall invertase	Similar to cell wall invertase (EC 3.2.1.26)	Han et al., 2016
	*OsSGR1*	9	20,868,846–20,871,077	*Os09g0532000*	Stay-green protein	Senescence-inducible chloroplast protein, activation of the chlorophyll-degrading pathway during leaf senescence	Han et al., 2016
	*OsAPO1/FBX202*	6	27,480,082–27,481,453	*Os06g0665400*	Aberrant panicle organization	F-box protein, inflorescence form, loading resistance and grain yield	Han et al., 2016
	*OsFBX94*	3	16,171,366–16,172,869	*Os03g0399400*	Aberrant panicle organization	Cyclin-like F-box domain containing protein	Han et al., 2016
	*OsFBX258*	7	25,488,479–25,489,870	*Os07g0617700*	Aberrant panicle organization	Cyclin-like F-box domain containing protein	Han et al., 2016
	*OsEND93-1*	6	2,208,762–2,209,556	*Os06g0142350*	Early nodulin-like protein	Early nodulin-like protein	Han et al., 2016
	*OsEND93-2*	6	2,199,361–2,200,466	*Os06g0142200*	Early nodulin-like protein	Early nodulin-like protein	Han et al., 2016
	*OsEND93-3*	6	2,212,615–2,213,482	*Os06g0142400*	Early nodulin-like protein	Early nodulin-like protein	Han et al., 2016
	*SGR*	9	20,868,846–20,871,077	*Os09g0532000*	Stay-green protein	Senescence-inducible chloroplast protein, activation of the chlorophyll-degrading pathway during leaf senescence	Park et al., 2007

**TABLE 6:** Detailed description of network genes associated with nitrogen use efficiency in wheat crop.

Category	Gene	Chr	Location	Locus name	Gene family	Phenotypic description	References
**Transporters**	*TaNPF1.1*	3A	540,654,271–540,656,804	*TraesCS3A02G304400*	Nitrogen transporter	Low-affinity transporter	Buchner and Hawkesford (2014)
	*TaNPF2.1*	5A	3,085,412–3,088,853	*TraesCS5A02G004400*	Nitrogen transporter	Low-affinity transporter	Buchner and Hawkesford (2014)
	*TaNPF2.2*	5A	34,980,804–34,986,700	*TraesCS5A02G037900*	Nitrogen transporter	Low-affinity transporter	Buchner and Hawkesford (2014)
	*TaNPF2.3*	2A	17,869,278–17,871,731	*TraesCS2A02G045500*	Nitrogen transporter	Low-affinity transporter	Buchner and Hawkesford (2014)
	*TaNPF2.4*	3A	660,436,466–660,444,074	*TraesCS3A02G418700*	Nitrogen transporter	Low-affinity transporter	Buchner and Hawkesford (2014)
	*TaNPF6.1*	6A	486,547,388–486,550,355	*TraesCS6A02G263500*	Nitrogen transporter	Low-affinity transporter	Buchner and Hawkesford (2014)
	*TaNPF6.2*	1A	373,766,258–373,768,702	*TraesCS1A02G210900*	Nitrogen transporter	Low-affinity transporter	Buchner and Hawkesford (2014)
	*TaNPF6.5*	1A	14,519,757–14,525,659	*TraesCS1A02G031300*	Nitrogen transporter	Low-affinity transporter	Buchner and Hawkesford (2014)
	*TaNPF6.6*	5A	599,204,895–599,208,619	*TraesCS5A02G409600*	Nitrogen transporter	Low-affinity transporter	Buchner and Hawkesford (2014)
	*NRT1 PTR*	7A	169,020,411–169,025,550	*TraesCS7A02G206400*	Nitrate transporter	Protein NRT1/PTR FAMILY 5.1 G	Léran et al., 2014
	*TaNPF7.1*	6AL/BL/DL	486,547,388–486,550,355	*TraesCS6A02G263500*	Nitrogen transporter	Low-affinity transporter	Buchner and Hawkesford (2014)
**Amino acid biosynthesis**	*TaGS1*	6AL/BL/DL	–	*DQ124209;DQ124210; DQ124211*	Glutamine synthase	Ammonia channeling for glutamine formation	Buchner and Hawkesford (2014)
	*TaGS2*	2AL/BL/DL	–	*DQ124212;DQ124213; DQ124214*	Glutamine synthase	Ammonia channeling for glutamine formation	Buchner and Hawkesford (2014)
	*TaGSe*	4AS/BS/DS	–	*AY491970;AY491971*	Glutamine synthase	Ammonia channeling for glutamine formation	Buchner and Hawkesford (2014)
	*TaGSr*	4AS/BS/DS	–	*AY491968;AY491969*	Glutamine synthase	Ammonia channeling for glutamine formation	Buchner and Hawkesford (2014)
	*TaGDH2*	2AL/BL/DL	–	*AK331666;TC266053*	Glutamate dehydrogenase 2	Deamination of glutamate to alpha-ketoglutarate	Buchner and Hawkesford (2014)
**Other genes**	*Rbcs*	2A	171,076,784–171,079,172	*TraesCS2A02G198700*	Rubisco gene	Gene for the small subunit of the chloroplast photosynthetic enzyme rib- ulose-1,5-bisphosphate carboxylase/oxygenase (Rubisco)	Laperche et al., 2006

Nitrate is the most common form of nitrogen present in soil which is transported in plants actively with the help of nitrate transporters. These nitrate transporters are encoded by NRT families. Firstly, these families were reported in Arabidopsis and were categorized into three subfamilies, i.e., NRT1 family, whose members are low-affinity transporters, and the NRT2/NRT family which primarily encodes high-affinity transporters (Plett et al., 2010). This information was used to find the orthologs of NRT transporter genes in cereal crops by using the reciprocal best hit (RBH) approach. It was observed that within cereals there is variability in gene number and family structure (Plett et al., 2010). Cereals express an additional *NRT1.1* ortholog and devoid of *NRT1.6*/*NRT1.7* in comparison to Arabidopsis. NRT2 family needs special focus in cereals for its functional analysis as this gene in grasses shows significant genetic distance. In rice, there are a total of four high-affinity NRT2 transporters (Table 5; Li et al., 2016). Among these four, two (*OsNRT2.1* and *Os-NRT2.2*) genes have high similarities to NRTs in monocots, while *OsNRT2.3* and *OsNRT2.4* are more closely related to the Arabidopsis NRT2 (Cai et al., 2008). Rather than the above-described gene variants, *OsNRT2.3* has further two subtypes, i.e., *OsNRT2.3a* and *OsNRT2.3b*. The overexpression of *OsNRT2.3b* is known to have a significant role in high grain yield and NUE in rice (Fan et al., 2016). It is established that in common wheat approximately 16 low-affinity nitrate transporter NPFs are expressed which are homologous to Arabidopsis NPFs (Buchner and Hawkesford, 2014). The expression of a particular transporter in wheat depends upon the nitrogen status of the plant and soil. NPF wheat genes have been reported to be regulated by plant nitrogen status, which suggests that nitrogen metabolism is the main regulator for genes involved in nitrate transport. Nitrate transporters are the main players in nitrogen uptake in most plants as nitrate is a precursor for N present in the soil, but in certain cases, ammonia is the predominant form in the soil. As in the case of rice when grown in paddy fields, ammonium ions (NH_4_^+^) are a major source of nitrogen. In such conditions, genes for ammonium transports, i.e., high-affinity transporter systems (HATS), for ammonia are expressed in roots (Tabuchi et al., 2007).

It is reported that HATS for ammonium transport in roots belong to the ammonium transporter/Rhesus-type/methylamine permease (AMT/Rh-type/MEP) protein family. The ammonium transporter system is well established in rice. It is known that there are 10 members of the AMT family, which are broadly classified as high and low-affinity transporters. Among the ten members, three *OsAMT1* family members fall under the category of high-affinity transporters, whereas three *OsAMT2*, *three OsAMT3 members*, and one *OsAMT4* member are components of low-affinity transporters (Loqué and Wirén, 2004). Although all sets of genes are present in rice, their expression varies, some are constitutively expressed in the roots and shoot while some are members of induced genes which are triggered after ammonium exposure or a decrease in plant N content (Kumar et al., 2003; Sonoda et al., 2003; Suenaga et al., 2003).

Nitrogen uptake is followed by nitrogen assimilation. It is a crucial metabolic step that regulates the grain yield and ultimately NUE. The glutamine synthetase (GS)/glutamate synthase (GOGAT) cycle is majorly involved in nitrate assimilation in the form of amino acids. The overexpression of genes encoding enzymes involved in the GS/GOGAT cycle is directly correlated with enhanced growth rate, biomass, and yield in rice. Especially overexpression of OsGS1 is reported to have a positive effect on grain yield under the influence of nitrate assimilation (Brauer et al., 2011). Three different forms of GS are reported in rice. Wheat is reported to have increased activity of GS1 especially in leaves which ultimately leads to accumulation of nitrogen in grains and also enhanced grain dry matter. Rice is known to have a small family of GS and GOGAT enzymes present in different cellular locations. Among variable isoforms of GS and GOGAT the cytosolic GS1;2 and the plastidic NADH-GOGAT1 are involved in ammonium ion assimilation in roots. It is reported that in conditions with high N content, overexpression of the GS1 gene enhances the nitrogen harvest index and NUE but no change in NUE was observed in a nitrogen-deficient environment.

Among cereals, maize has a C4 system so it has an enhanced capacity to assimilate and metabolize carbon and nitrogen. Expression of NAD-malic enzymes in C4 plants is responsible for enhanced nitrogen assimilation as compared to C3 plants. It is established from knock-out studies that overexpression of genes for two isoforms of enzymes, i.e., *Gln1-3* and *Gln1-4* genes in the maize leads to an increase in kernel number (Martin et al., 2006; Sun et al., 2018). Therefore, the gene for nitrogen assimilation plays a major role in kernel yield. Similar studies were conducted in barley where an extra copy of the *HvGS1-1* gene was expressed which was reported to enhance the GS1 enzyme activity and such lines displayed high NUE and grain yield as compared to wild-type plants (Gao et al., 2018).

The last step in nitrogen use efficiency is the remobilization of nitrogen toward seeds during maturity. Monocots, dicots, C3, and C4 plants share a common mechanism for nitrogen remobilization (Masclaux-Daubresse et al., 2010). Among different amino acids, asparagine and glutamine are the common transport form and its concentration increase in phloem sap during senescence of leaves for nitrogen remobilization to reproductive tissue. In durum wheat asparagine synthetase encoding genes (AsnS1) are crucial for nitrogen remobilization from flag leaf to developing grains (Curci et al., 2018). Similarly, in rice, the growing panicle derives approximately 80% of the nitrogen from the senescing organs and reaches reproductive organs through the phloem. Nitrogen is majorly transported in the phloem sap in the form of glutamine. Two enzymes GS and GOGAT are essential for nitrogen remobilization and reutilization in senescing and developing organs, respectively (Tabuchi et al., 2007). In rice, it is observed that GS1-1 is crucial for the remobilization process, whereas NADH-GOGAT1 is involved in the reutilization of transported glutamine in growing tissues (Hayakawa et al., 1994; Tabuchi et al., 2007). In maize, wheat, and barley, the grain nitrogen content is correlated with flag leaf senescence, which seems to play an important role in nitrogen availability for grain filling (Martin et al., 2006; Uauy et al., 2006). High yield is reported to be affected by leaf senescence, as delayed leaf senescence is responsible for prolonged photosynthesis, which improved the grain yield. However, the delayed leaf senescence was reported to decrease nitrogen remobilization efficiency and grain protein content (Masclaux-Daubresse et al., 2010).

### Transcription Factors Involved in NUE

The major switches in the plant regulatory networks are transcription factors and like several metabolic processes NUE is crucially dependent on coordinated transcription factors (Figure 6 and Tables 5, 6; Spitz and Furlong, 2012). Transcription factors involved in lateral root growth in response to nitrate belong to MADS-box and ANR1 is a member of the transcription factors reported in Arabidopsis (Zhang, 1998). These transcription factors initiate the signaling pathway of *NRT1.1* (Remans et al., 2006). Another family of transcription factors involved in nitrogen metabolism is the NLP (NIN-like protein) family of transcription factors (Konishi and Yanagisawa, 2013; Marchive et al., 2013). These transcription factors are reported to interact with NLP genes, including TCP20 (teosinte branched1/cycloidea/proliferating cell factor1-20) (Guan et al., 2017). This interaction is important for lateral root growth in response to nitrate (Guan et al., 2014; Xu et al., 2016). BT1 and BT2 (bric-abrac/tramtrack/broad) form the third major family of transcription factors which act on multiple genes to form a network for nitrate assimilation. System biology approaches were used to discover the web of transcription factors involved in NUE. Functional analysis indicated that the transcription factor which actively regulates NUE in Arabidopsis have orthologs in cereals especially rice (Araus et al., 2016). The transcription factors belonging to the DOF (DNA-binding with one finger) and bHLH (helix loop helix) families are actively involved in NUE is rice (Table 5). These transcription factors are reported to be involved in various biological processes, such as tissue differentiation and hormone signaling (Noguero et al., 2013). A report suggests that enhanced expression of the DOF1 gene in rice increases N assimilation and plant growth under low-N conditions (Kurai et al., 2011). DOF family transcription factors are reported to control ammonium uptake by inducing genes of the ammonium transporter family in roots of rice (Wu et al., 2017). Alongside this, in wheat the DOF1.3 gene was overexpressed under stress conditions such as nitrogen starvation (Curci et al., 2017). A total of 170 unique genes encoding transcription factors belonging to the different families, including bHLH, MYB, bZIP, C2C2-Dof, TERF, WRKY, NF-Y, NAC, AUX/IAA, and the auxin-modulated ARF, etc., displayed differential expression between nitrogen-stressed and control durum wheat tissues.

### miRNA Involved in Different Aspects of NUE

miRNAs are reported to play important roles in NUE along with several transcription factors. The miRNA169 family is reported to regulate the expression of genes for nitrogen transport under low nitrogen conditions. This family of miRNA is broadly studied among cereals, as it is reported in maize, miR169 expression decreases in N-deficient plants (Zhao et al., 2012). Furthermore, several new miR169 family members are reported to express in durum wheat which responds to nitrogen-deficient conditions (Zuluaga et al., 2017, 2018). The conserved ttu-miR169h and ttu-miR169c at the seedling and grain filling stages, respectively, and the newly identified ttu-novel-61 belonging to the miR169 family, were downregulated in both stages of durum wheat plants subjected to nitrogen starvation in both the roots and leaves. These miRNAs are known to negatively regulate the CCAAT box-binding transcription factors in several tissues which influence the NUE-related genes in durum wheat plants (Zuluaga et al., 2017). Through miRNA studies, several transcription factors and genes are revealed to have an important role in enhancing NUE, for example, degradome libraries and sequencing of miRNAs among maize seedlings revealed that there are 99 loci categorized into 47 miRNA families, 9 of which are paralog to miR169, miR171, and miR398 (Zhao et al., 2012). Besides, eight miRNA families showed differential expression under nitrogen-deficient conditions and the target analysis proposed a role of newly identified miRNA target genes in a wide range of metabolic processes and cellular responses (Zhao et al., 2012). Recently, a study involving degradome sequencing and small RNA together with target gene validation showed that two new putative miR169 species (miRC10 and miRC68) may play a key role in low nitrogen adaptation of maize seedlings (Zhao et al., 2013b). mir164a and mir164b are reported to have a specific role in nitrogen remobilization. The miR164 family is reported to influence NAC transcription factors and several studies were conducted to define the relation between miR164 and NAC regulation among cereals. The NAM-B1 gene in bread wheat was reported as a NAC transcription factor affecting the grain nutrient concentration (Waters et al., 2009) in addition to increasing the remobilization of nutrients from leaves to developing grains in wild wheat. Further, zma-miR164 in maize was downregulated in leaves after severe nitrogen stress treatment (Xu et al., 2011). The regulation of NAC genes by miR164 in cereals may maintain the nitrogen remobilization from leaves to seeds under low nitrogen conditions.

The expression of variable miRNAs in rice among low nitrogen tolerant and sensitive genotypes through a microarray showed differential expression of a total of 32 miRNAs between two genotypes including miR164 and another 7 miRNAs. Six miRNAs, viz., miR156, miR164, miR820, miR528, miR821, and miR1318 and four miRNAs, viz., miR164, miR528, miR167, and miR168 showed differential expression in leaves and roots, respectively (Nischal et al., 2012). The identified miRNAs were predicted to control genes encoding for the proteins and the transcription factors associated with stress responses or metabolic processes. Many miRNAs are reported to be involved in stress response in plants. Although they do not have a direct role in NUE, their involvement in stress response makes them important while considering several factors affecting NUE. In the roots of maize, under low NO_3_^–^ conditions miR528a,b, and miR528a^∗^,b^∗^ were repressed suggesting their role in integrating NO_3_^–^ signals into root developmental changes (Trevisan et al., 2012). Moreover, Zma-miR528a,b family members showed downregulation in maize roots and leaves of seedlings exposed to nitrogen deficiency (Zhao et al., 2012). It was reported that increased expression of rice miR528 was subsequently associated with an increase in total nitrogen accumulation, plant biomass, and chlorophyll synthesis (Yuan et al., 2015). miR528 in rice is known to be involved in enhancing N-mediated tillering by inhibiting auxin signaling in axillary buds. Along with it, Osa-miR393 is another class of miRNA expressed in rice acting as a regulator of OsTB1 and OsAFB2 genes (Li et al., 2016). Under nitrogen deficit conditions, TaMIR1129, TaMIR1118, and TaMIR1136 were reported to be upregulated, whereas TaMIR1133 was downregulated in roots in wheat. The expression of some of these miRNAs was inversely correlated with the concentration and duration of nitrogen application (Zhao et al., 2013a). TaMIR2275, another common wheat miRNA, showed gradual upregulation during nitrogen starvation, while the expression of miRNA was progressively restored upon nitrogen recovery treatment. Overexpression of TaMIR2275 produced plants with increased nitrogen accumulation and biomass, while the reverse was observed in the knockdown mutants. Consequently, several classes of miRNAs are involved in nitrogen metabolism by affecting multiple processes associated directly or indirectly with NUE. Overall, it is essential to understand the precise network of miRNA expression and interaction to completely channelize the mechanism underlying NUE.

**FIGURE 7:**
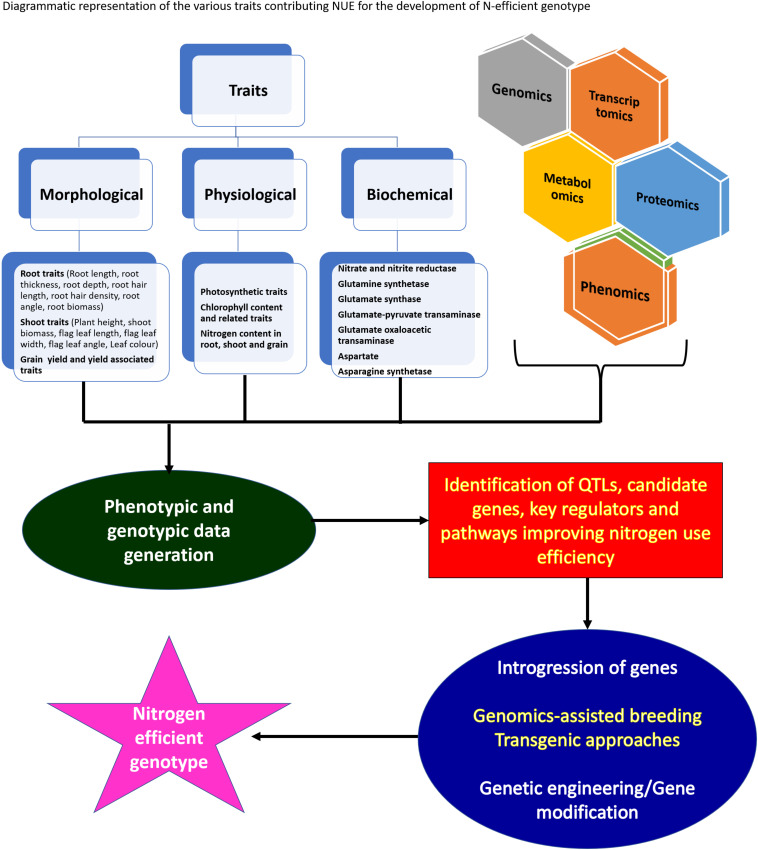
Schematic representation of the various traits, QTL, and candidate genes contributing to the development of nutrient efficient genotypes. The pipeline for identification and deployment of traits/QTL/gene/regulatory factors associated with nitrogen use efficiency. The identified traits and donors can be used to develop suitable mapping populations to be further used in mapping of genomic regions and identification of candidate genes or key regulators associated with trait of interest using recent biotechnological approaches such as phenomics, genomics, transcriptomics, metabolomics, and proteomics. The deployment of identified QTL/gene/regulatory factors in genomics-assisted breeding programs and their functional characterization employing transgenic and genetic engineering approaches.

The identification of suitable traits, QTL, and candidate genes underlying QTL may provide new opportunities for the introgression of these QTL and genes into elite genetic backgrounds contributing to the development of nutrient efficient varieties (Figure 7).

## Conclusion

Excessive use of nitrogen fertilizers to boost the grain yields of cereal crops is a major cause of water, soil, and air pollution as well as greenhouse gas emissions. It has an economic impact globally due to the high production costs of nitrogen fertilizer. The challenge in improving NUE in cereal crops is achieving both high yield and high nitrogen use efficiency (NUE) simultaneously. Therefore, improving nitrogen use efficiency is very important for environmentally friendly and profitable crop production. The ultimate goal of improving our understanding of agronomic management, suitable traits, QTL, genes, and the mechanisms and functions of genes associated with nitrogen use efficiency is to enhance crop production and productivity. The careful selection of diverse genotypes, exploitation of natural variation, exploring root architecture, high-throughput precise phenotyping, standardized field trials, new techniques for efficient fertilizer application, appropriate field management practices, and identification of new QTL/genes/nitrogen transporters and signaling molecules could be helpful to reduce fertilizer consumption in the near future. The challenge here is to identify consistent genomic regions and molecular regulators interacting at several nodes in the gene network to act as the key component in nitrogen metabolism. The improvement in basic research in combination with agronomical, marker-aided molecular breeding and biotechnological strategies will help to achieve higher nitrogen use efficiency in cereal crops. The compiled information on QTL in the present review can be used further in metaQTL analysis to study the congruency of the identified regions associated with particular traits of interest.

## Author Contributions

NS: conceptualization, funding acquisition, resources, and supervision. NS, MS, AK, DD, and JS: literature search. NS and MS: writing–original draft. NS and PC: critical review and editing. All authors contributed to manuscript revision, read, and approved the final version of the manuscript.

## Conflict of Interest

The authors declare that the research was conducted in the absence of any commercial or financial relationships that could be construed as a potential conflict of interest.
